# Multifrequency Time‐Dependent Deep Image Prior for Real‐Time Free‐Breathing Cardiac Imaging

**DOI:** 10.1002/nbm.70114

**Published:** 2025-08-04

**Authors:** Jesse I. Hamilton, Gastao Cruz, William Truesdell, Prachi Agarwal, Imran Rashid, Nicole Seiberlich

**Affiliations:** ^1^ Department of Radiology University of Michigan Ann Arbor Michigan USA; ^2^ Department of Biomedical Engineering University of Michigan Ann Arbor Michigan USA; ^3^ School of Medicine Case Western Reserve University Cleveland Ohio USA; ^4^ Harrington Heart and Vascular Institute University Hospitals Cleveland Ohio USA

**Keywords:** cardiac imaging, deep learning, manifold, non‐Cartesian, real‐time

## Abstract

The aim of this study is to enable high temporal resolution functional cardiac imaging without breathholds or electrocardiogram (ECG) gating. Real‐time MRI is essential for assessing heart function in patients with limited breathhold capacity or arrhythmias that preclude breathheld ECG‐gated cine scans. The Time‐Dependent Deep Image Prior (Time‐DIP) method is a promising reconstruction for dynamic MRI, combining a nonlinear manifold with zero‐shot deep learning. However, while Time‐DIP has been demonstrated for breathheld cine imaging, it employs a helical manifold that assumes quasi‐periodic motion and thus may not be suitable for *free‐breathing* real‐time scans, particularly in arrhythmia patients. This study proposes a Multifrequency Time‐DIP technique to extend this framework to free‐breathing real‐time cardiac imaging. First, a “multifrequency manifold” is introduced that parameterizes time using multiple sinusoids spanning various frequencies, enabling dynamic imaging without assuming motion periodicity. Second, joint estimation of coil sensitivities using zero‐shot deep learning is used to improve the reconstruction of multichannel data. Simulations and scans of healthy subjects and patients, including those with arrhythmias, were performed using a 2D free‐breathing ungated golden angle spiral bSSFP sequence. Image quality and left ventricular (LV) functional measurements were compared to real‐time scans reconstructed with compressed sensing and the original Time‐DIP implementation, as well as conventional breathheld ECG‐gated cine scans. Multifrequency Time‐DIP outperformed other real‐time techniques in simulations of various motion scenarios. In vivo scans using Multifrequency Time‐DIP exhibited reduced aliasing artifacts, achieving temporal resolutions as high as a single TR (4.2 ms/frame), with no significant differences in LV functional measurements compared to conventional scans. While conventional scans had better edge sharpness and image contrast scores, Multifrequency Time‐DIP exhibited overall higher image quality metrics among real‐time scans. In conclusion, a generalized Time‐DIP reconstruction was shown to enable high temporal resolution free‐breathing real‐time cardiac imaging in healthy subjects and patients, including those with arrhythmias.

## Graphical abstract

This study introduces a Multifrequency Time‐Dependent Deep Image Prior reconstruction that combines a multifrequency manifold, comprised of multiple sinusoids spanning a range of frequencies, with zero‐shot deep learning. Real‐time free‐breathing cardiac imaging with a temporal resolution as high as a single TR is demonstrated in healthy subjects and arrhythmia patients.

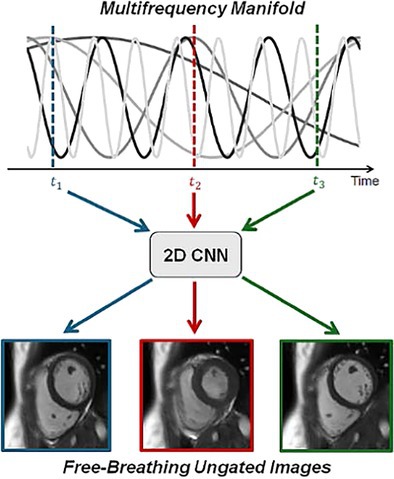

**Abbreviations**
bSSFP: balanced steady‐state free precession
CNN: convolutional neural network
CS: compressed sensing
CV: coefficient of variation
DIP: deep image prior
ECG: electrocardiogram gating
EDV: end‐diastolic volume
EF: ejection fraction
ESPIRiT: eigenvector‐based iterative self‐consistent parallel imaging reconstruction
ESV: end‐systolic volume
FOV: field of view
LV: left ventricle
PVC: premature ventricular contractions
RMSE: root mean square error
ROI: region of interest
SToRM: smoothness regularization on manifolds
Time‐DIP: time‐dependent deep image prior
TR: repetition time

## 1 Introduction

Cine MRI is the gold standard for quantifying left ventricular (LV) volume and ejection fraction (EF), key metrics for evaluating cardiovascular health [1]. Traditional 2D segmented Cartesian cine imaging relies on retrospective electrocardiogram (ECG) gating and requires patients to perform multiple breath holds over several minutes to achieve full LV coverage, which can be particularly challenging for patients with limited breath hold capacity. Furthermore, ECG gating often fails in the presence of arrhythmias, leading to motion artifacts that compromise image quality. These limitations have motivated interest in real‐time scans, which capture images at sufficiently high temporal resolution to visualize cardiac motion without the need for breath holds and ECG synchronization.

Real‐time cardiac MRI typically requires high acceleration factors. While established reconstruction methods, including parallel imaging [2, 3, 4, 5, 6], compressed sensing [7, 8], and low‐rank modeling [9, 10], have been successfully applied to real‐time cardiac MRI, each faces inherent limitations. Parallel imaging performance is determined by the number and geometry of receiver coils, which often limits these methods to low acceleration factors. Compressed sensing (CS) leverages the sparsity of images in a transform domain—often exploiting temporal sparsity in dynamic scans—and can generally achieve higher acceleration factors than parallel imaging. CS techniques, including advanced methods such as XD‐GRASP that leverage sparsity along cardiac and respiratory motion dimensions, have shown promising results for free‐breathing cine imaging [11]. However, CS methods remain fundamentally constrained by sparsity assumptions, which may prove overly restrictive in the presence of irregular or complex motion dynamics, potentially leading to residual aliasing artifacts or blurring. Similarly, low‐rank subspace techniques, which model dynamic images as linear combinations of a small number of basis functions, may fail to accurately represent free‐breathing real‐time cardiac scans with extensive or irregular motion.

Nonlinear manifold‐based reconstructions have emerged as a promising approach for accurately recovering respiratory and cardiac motion dynamics in accelerated real‐time scans [12, 13, 14]. Instead of enforcing temporal sparsity directly on the images, as in compressed sensing, these methods exploit the smoothness of a low‐dimensional manifold by modeling temporally adjacent frames as nearby points on the manifold, as in the SmooThness Regularization on Manifolds (SToRM) technique [12]. Moreover, several groups have shown that manifold reconstructions can be augmented with deep learning to increase the spatiotemporal resolution and shorten scan times [15].

A challenge with traditional deep learning approaches that employ supervised learning is the need to acquire high‐quality reference (i.e., fully‐sampled) data for training. This training data can be time‐consuming and costly (and in some cases impossible) to obtain, particularly when seeking to perform real‐time scans in the presence of motion. As an alternative, deep learning methods that employ zero‐shot learning may be more suitable for real‐time cardiac applications, as they are trained in a scan‐specific manner using only the undersampled k‐space data from a single scan. However, zero‐shot learning also requires careful training strategies, such as early stopping or dropout regularization, to mitigate the risk of overfitting to noise and aliasing artifacts [16].

Reconstructions combining manifolds with zero‐shot deep learning have been successfully applied to real‐time cardiac imaging [17]. One notable example is the Time‐Dependent Deep Image Prior (Time‐DIP) [18], which parameterizes the time dimension of a dynamic scan using a low‐dimensional manifold that is subsequently input into a convolutional neural network (CNN) to reconstruct time‐resolved images. However, prior studies with Time‐DIP were limited to ungated *breath‐held* cine imaging in healthy subjects, and the manifold design was tailored to individual datasets based on the number of heartbeats captured during each scan. Most importantly, the specific manifold design (a helix) was biased toward quasi‐periodic cardiac motion, which may introduce errors when imaging arrhythmia patients or during *free‐breathing* scans with deep or irregular breathing patterns.

This study aims to extend the Time‐DIP reconstruction to free‐breathing real‐time cardiac imaging and to evaluate its performance in healthy subjects and patients, including those with arrhythmias. Two key modifications are proposed. First, a novel multifrequency manifold design is introduced that encodes temporal variations using sinusoids spanning a range of frequencies. The manifold does not require fine‐tuning for individual scans and makes no assumptions about motion periodicity. Second, joint estimation of coil sensitivities is integrated within the zero‐shot deep learning framework to improve the reconstruction of multichannel data. The proposed reconstruction, termed a Multifrequency Time‐Dependent Deep Image Prior, is evaluated in simulations and free‐breathing real‐time golden angle spiral bSSFP scans of 10 healthy subjects and 10 patients, including those with arrhythmias, undergoing a clinical MRI exam for suspected cardiomyopathy. Image quality metrics and LV functional measurements are compared to real‐time spiral scans using compressed sensing and the original Time‐DIP reconstruction, as well as Cartesian breathheld ECG‐gated cine scans.

## 2 Methods

### 2.1 Overview of the Time‐Dependent Deep Image Prior

This section provides a brief overview of the Time‐DIP reconstruction proposed by Yoo et al. [18]. In this approach, the temporal dimension of a dynamic image series is modeled using a low‐dimensional manifold. A three‐dimensional helix was found to be an appropriate manifold design for breathheld ungated cine imaging, where the number of twists in the helix (denoted by p) is adjusted to approximately match the number of heartbeats in the scan. The number of heartbeats can be estimated by filtering the signal at the center of k‐space for data sampled using a non‐Cartesian trajectory. The helix manifold reflects the quasi‐periodic nature of cardiac motion and is defined as follows, where T is the total number of image frames.

(1) $$z_{t}=\left[\cos\left(\frac{2\mathrm{\pi tp}}{T-1}\right),\sin\left(\frac{2\mathrm{\pi tp}}{T-1}\right),\frac{t}{T-1}\right]$$

As shown in Figure S1, image reconstruction is performed by sampling the manifold at a specific time frame t. The manifold sample, $z_{t}$, is input to a fully connected network, denoted by g (also termed MapNet in the original study), which transforms the manifold into a higher‐dimensional latent space. The output of this step is then processed by a CNN (denoted by h) to generate a complex‐valued image frame $x_{t}$.

(2) $$x_{t}=\left(h\circ g\right)\left(z_{t}\right)$$

The processes of manifold sampling and CNN image generation are repeated for all T frames to reconstruct the entire dynamic image series. As described later, network training is performed in a scan‐specific manner by enforcing consistency between the reconstructed images and undersampled k‐space data from a single scan, with no need for fully‐sampled reference data.

### 2.2 Multifrequency Manifold for Time‐DIP

**FIGURE 1:**
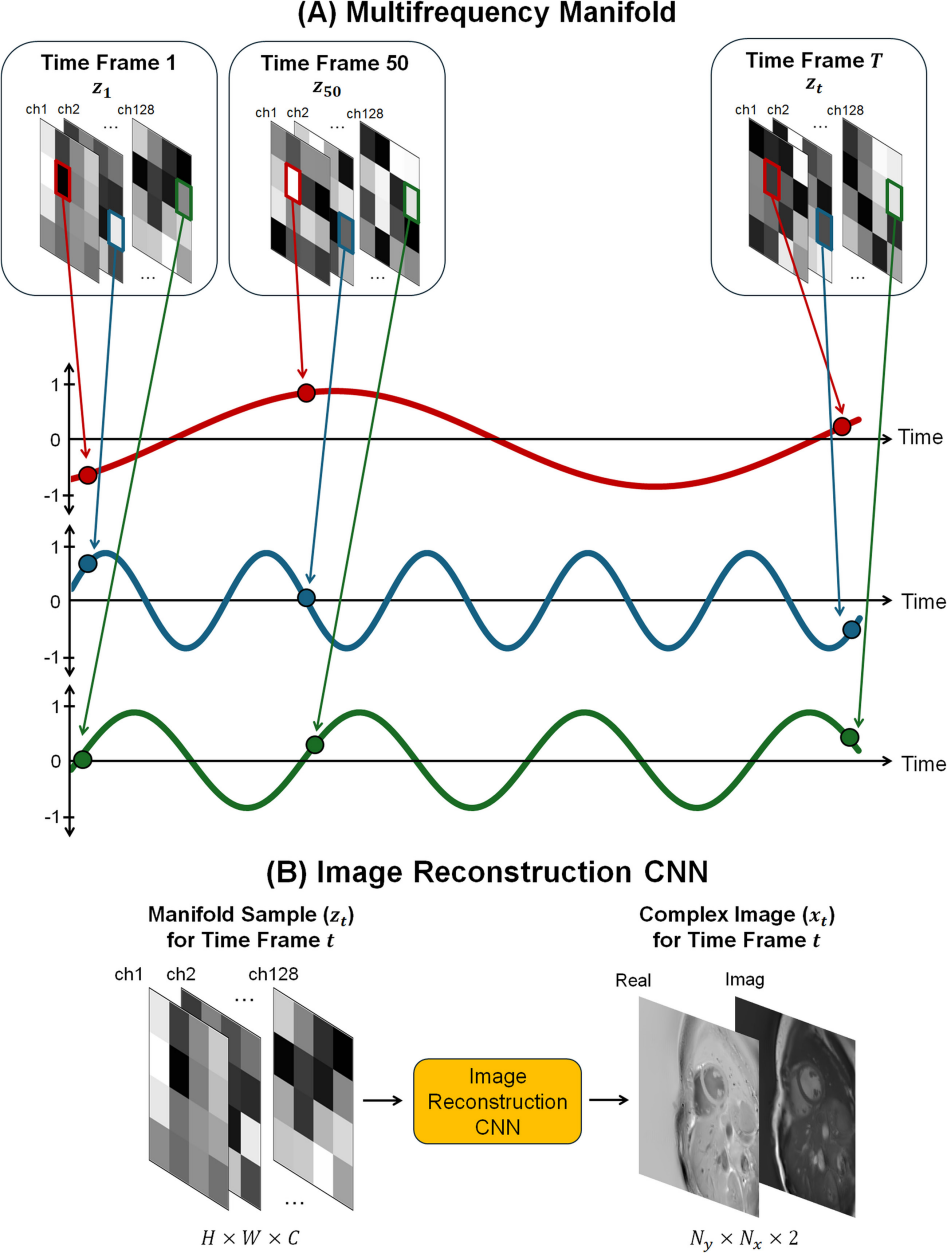
Time‐dependent deep image prior with a multifrequency manifold. (A) The multifrequency manifold parameterizes the time dimension of a dynamic scan using multiple sinusoids spanning a range of frequencies. The manifold is sampled for a specific time frame t, producing a tensor $z_{t}$ with dimensions H×W×C (height × width × channels). For clarity, the diagram shows a height and width of 4 × 4, although the actual size was 8 × 8. At each coordinate $\left(h,w,c\right)$, the value of $z_{t}\left(h,w,c\right)$ evolves sinusoidally over time with a randomly selected frequency $f\left(h,w,c\right)$ and initial phase $\phi \left(h,w,c\right)$. Three coordinates are highlighted showing examples of low‐ (red), medium‐ (green), and high‐frequency (blue) sinusoidal variations. (B) Dynamic images are reconstructed by sampling the manifold for each time frame, yielding a tensor $z_{t}$ that is input into the Image Reconstruction CNN to produce a complex image $x_{t}$.

The helical manifold described above is designed to capture the quasi‐periodic nature of normal cardiac motion; however, this assumption does not hold in the presence of arrhythmias. Additionally, this manifold does not explicitly account for additional motion sources, such as respiration during free‐breathing scans. To address these limitations, this study proposes a novel manifold design composed of sinusoids spanning a range of frequencies, which we term a “multifrequency manifold” (Figure 1).

As in the original Time‐DIP approach, this manifold is sampled at a specific time point t, producing a tensor $z_{t}$ with dimensions of H×W×C (height × width × channels), which is input to a CNN (referred to as the Image Reconstruction CNN or $\theta_{\text{image}}$) to produce a complex‐valued image $x_{t}$.

(3) $$x_{t}=\theta_{\text{image}}\left(z_{t}\right)$$

Note that C refers to the number of network channels, not the number of MRI receiver coils. In this study, the manifold dimensions are fixed at 8×8×128 (the choice of these values is justified in Figure S2). At each coordinate $\left(h,w,c\right)$ within the manifold, the value of $z_{t}$ evolves over time according to a sinusoidal function, with each coordinate randomly assigned a unique frequency $f\left(h,w,c\right)$ within specified bounds and an initial phase $\phi \left(h,w,c\right)$. Mathematically, the multifrequency manifold is described as follows:

(4) $$z_{t}\left(h,w,c\right)=\sin\left[2\mathrm{\pi f}\left(h,w,c\right)t+\phi \left(h,w,c\right)\right]$$

Lower frequency sinusoids facilitate the recovery of slower motion dynamics, such as respiration, since temporally adjacent time points are encoded with similar values. Conversely, higher‐frequency sinusoids enable the recovery of faster dynamics, such as cardiac motion, as well as abrupt and aperiodic motion, since temporally adjacent time points are encoded with very different values. The entire manifold is comprised of a spectrum of different frequencies, totaling 8*8*128 or 8192 unique frequencies given the manifold dimensions in this study. This design grants flexibility for representing diverse respiratory and cardiac motion dynamics and, unlike the original helical manifold, does not require scan‐specific adjustments (i.e., tuning the number of helix twists to match the number of heartbeats in the scan).

The proposed Multifrequency Time‐DIP reconstruction omits the fully connected network (MapNet) used in the original Time‐DIP method, as preliminary studies with the multifrequency manifold found that omitting MapNet yielded lower errors (Table S1). The Image Reconstruction CNN architecture is similar to that used in the original work (Figure S3) and consists of multiple blocks of 3 × 3 convolutions, nonlinear ReLU activations, and 2 × 2 nearest neighbor upsampling operations. The CNN outputs an image frame $x_{t}$ with dimensions $N_{y}\times N_{x}\times 2$, where $N_{y}$ and $N_{x}$ correspond to the spatial dimensions (e.g., 128 × 128), and the two channels represent real and imaginary parts.

### 2.3 Joint Estimation of Coil Sensitivities

Joint estimation of coil sensitivities has been shown to improve image quality and enable higher acceleration factors in parallel imaging (JSENSE) [19] and model‐based (NLINV) [20] reconstructions, and extensions of these approaches using supervised deep learning have also been proposed [21]. Here, to improve the reconstruction of multichannel data, a strategy for jointly estimating coil sensitivities within the Time‐DIP framework is introduced. First, initial sensitivity maps, denoted $S_{0}$, are derived from the time‐averaged k‐space data using ESPIRiT [22]. The MRI receiver coil dimension is treated as the neural network channel dimension by reshaping the sensitivity maps to $N_{y}\times N_{x}\times 2N_{c}$, where $N_{c}$ is the number of receiver coils, with the factor of two accounting for the real and imaginary components. These reshaped maps are then input to a second network, termed the Sensitivity Map CNN or $\theta_{\text{coilmaps}}$. This network consists of four 3 × 3 convolutions with ReLU activations, followed by a final 1 × 1 convolution with a hyperbolic tangent activation (Figure S4). The output of this CNN is a refined estimate of the sensitivity maps, denoted S.

(5) $$S=\theta_{\text{coilmaps}}\left(S_{0}\right)$$

### 2.4 Zero‐Shot Training

**FIGURE 2:**
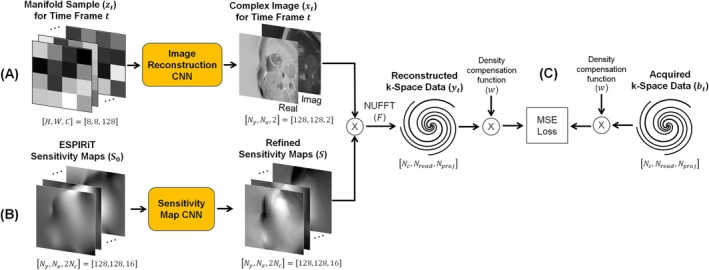
Zero‐shot network training. (A) In each training iteration, a single time frame t is randomly selected as a mini‐batch. The multifrequency manifold is sampled at this time point, producing a tensor $z_{t}$, which is input into the Image Reconstruction CNN to produce a complex image $x_{t}$. (B) In parallel, an initial estimate of the coil sensitivity maps $S_{0}$ (obtained using ESPIRiT) is processed by the Sensitivity Map CNN, yielding a refined estimate of the coil sensitivities, denoted S. (C) To ensure consistency with the acquired data, the reconstructed image $x_{t}$ is multiplied by the refined sensitivity maps S, and data are resampled along a spiral k‐space trajectory using the NUFFT. The MSE loss is calculated between the reconstructed ($y_{t}$) and acquired data ($b_{t}$) at the sampled k‐space locations after multiplication by the density compensation function (denoted by w). This loss is used to jointly update both CNNs.

Both CNNs are trained in a zero‐shot manner by enforcing consistency between the network‐generated images and sensitivity maps compared to the acquired (undersampled) k‐space data, without requiring any additional reference data for training (Figure 2). During each training iteration, a single time frame t is randomly selected as a mini‐batch. The multifrequency manifold is sampled at this time point and input to the Image Reconstruction CNN. In parallel, the initial ESPIRiT maps are refined by the Sensitivity Map CNN. The resulting image and sensitivity maps are multiplied, and data are resampled along the k‐space trajectory (here a spiral) using the non‐uniform fast Fourier Transform (NUFFT), denoted by F [23]. Both CNNs are updated simultaneously using a real‐valued mean squared error (MSE) loss, computed as the squared magnitude of the difference between the reconstructed data ($y_{t}$) and acquired data ($b_{t}$) at the sampled k‐space locations, after multiplication by the spiral density compensation function w.

(6) $$y_{t}=\mathrm{FS}x_{t}$$

(7) $$\underset{\theta_{\text{image}},\;\theta_{\text{coilmaps}}\;}{\arg\;\min}{\left|w\left(b_{t}-y_{t}\right)\right|}^{2}$$

A well‐known challenge with zero‐shot learning is the potential for overfitting to noise or undersampling artifacts given the lack of reference data. To address this issue, dropout regularization was applied after each convolutional layer in the CNNs [24], and early stopping was employed. Specifically, training was performed with a dropout rate of 5% and 300 epochs, where one epoch corresponded to a full pass over all time frames. These hyperparameters were selected based on simulations (Figure S5), where the inclusion of a small amount of dropout was found to reduce reconstruction errors and mitigate overfitting. Training was performed using an Adam optimizer with a learning rate of 0.001. The reconstruction was implemented in TensorFlow with a Keras backend and run on a high‐performance computing cluster using an NVIDIA A100 GPU with 8 GB memory allocated per job. Gridding of non‐Cartesian data was performed using the TensorFlow NUFFT library [25]. The reconstruction code is publicly available at: https://github.com/hamiljes/MultifrequencyTimeDIP.

### 2.5 Spiral Trajectory Design

All experiments used a variable density spiral designed for a 300 × 300 mm^2^ field‐of‐view (FOV), 128 × 128 matrix size, and 2.3 × 2.3 mm^2^ spatial resolution with a readout duration of 2.4 ms and TR of 4.2 ms [26]. The trajectory required 24 interleaves to fully sample the center 25% of k‐space and 48 interleaves for complete k‐space coverage. A spiral rewinder was designed to achieve balanced zeroth‐ and first‐order moments. A pseudo golden angle ordering scheme was used, in which the true golden angle was calculated for each TR, and from the 48 spiral interleaves available, the one nearest to this angle was selected. This approach allowed retrospective selection of the temporal resolution by binning any desired number of spiral interleaves per frame [27]. The density compensation function was calculated with the Michigan Image Reconstruction Toolbox (MIRT) [28] using an iterative method proposed by Pipe et al. [29].

### 2.6 Digital Cardiac Phantom Simulations

To evaluate the accuracy of the Multifrequency Time‐DIP reconstruction, simulations were performed using the XCAT digital cardiac phantom [30], which enables simulation of realistic respiratory and cardiac motion with user‐defined amplitudes and frequencies. First, time‐resolved images from a medial short‐axis slice were generated in which each pixel was assigned an integer label corresponding to a specific tissue type (blood, myocardium, etc.). Each tissue label was assigned a T_1_, T_2_, and proton density value representative of 1.5 T imaging. The resulting tissue property maps were then used to simulate dynamic images based on the steady‐state bSSFP signal equation, with golden angle spiral k‐space sampling performed using the NUFFT. To accurately model motion between TRs, each spiral interleaf was sampled from a distinct image frame. For example, to simulate an acceleration factor of R = 12 (4 interleaves per frame), 4 images were generated with incremental motion between them equivalent to one TR. One spiral interleaf was then sampled from each image, and the temporal average of these frames was used as the ground truth.

The first experiment compared Time‐DIP reconstructions using the original helix manifold and the proposed multifrequency manifold under the following motion scenarios: (1) breathheld imaging with a constant heart rate of 70 beats per minute (bpm), (2) free‐breathing imaging with a constant 70 bpm heart rate, a constant respiratory period of 3.5 s, and (3) free‐breathing imaging with a constant respiratory period and a cardiac arrhythmia, simulated using an ECG signal recorded from a patient with premature ventricular contractions (PVCs). In free‐breathing scans, displacements of 1.2 cm and 2 cm were simulated along anterior–posterior and superior–inferior directions, respectively, resulting in both in‐plane and through‐plane motion in a short‐axis view. For all scenarios, a 6‐s real‐time (ungated) golden angle spiral bSSFP scan was simulated with a TR of 4.2 ms, an acceleration factor of R = 8 (6 interleaves per frame, 25.2 ms/frame temporal resolution), and 238 total frames. Complex Gaussian noise with a standard deviation of 1% of the maximum DC signal was added to the simulated k‐space data. Coil sensitivities were not included in these simulations. Images were reconstructed using two methods. First, the original Time‐DIP approach with a helix manifold and MapNet was employed, hereafter referred to as *Helix Time‐DIP*, based on publicly available code from Yoo et al. [18]. The number of twists in the helix was matched to the number of heartbeats in the scan, determined by applying a bandpass filter (0.5‐ to 2‐Hz passband frequency) to the k‐space center signal. Second, the Multifrequency Time‐DIP reconstruction was used. The manifold was comprised of sinusoids with frequencies ranging from 0.05 to 20 Hz and omitting the MapNet, as described above. Reconstruction accuracy was assessed by calculating the normalized root mean square error (RMSE) averaged across all frames.

The second experiment evaluated how the maximum frequency bound of the manifold influenced reconstruction accuracy. XCAT data at an acceleration factor of R = 8 were simulated for free‐breathing scans under two conditions: a constant heart rate of 70 bpm and an arrhythmic case with PVCs. For this analysis, reconstructions were performed while keeping the minimum frequency bound fixed at 0.05 Hz and varying the maximum frequency bound from 0.2 to 20 Hz. The upper limit of 20 Hz was chosen based on the 25‐ms temporal resolution of the real‐time scan. At this frame rate, a sinusoid with a 50‐ms period (equivalent to 20 Hz) completes one full cycle between two consecutive frames, allowing it to effectively capture the highest temporal variations resolvable by the scan.

The third experiment assessed reconstruction accuracy at varying acceleration factors. A 6‐s free‐breathing scan under constant heart rate conditions was simulated for acceleration factors ranging from R = 4 (12 interleaves per frame, 50.4‐ms temporal resolution, 199 total frames) to R = 48 (1 interleaf per frame, 4.2‐ms temporal resolution, 1429 total frames). Reconstructions were performed using Multifrequency Time‐DIP, Helix Time‐DIP, and compressed sensing (CS) with temporal total variation regularization [11]. For the CS reconstruction, a range of regularization weights was tested at each acceleration factor, and the value yielding the lowest RMSE was selected to ensure a fair comparison. For the Multifrequency Time‐DIP reconstruction, the minimum frequency bound was fixed at 0.05 Hz, and the maximum bound was varied depending on the acceleration factor—from 10 Hz (period of 100 ms) at R = 4 to 120 Hz (period of 8 ms) at R = 48—to capture the highest temporal variations resolvable by the scan, as described above.

The fourth experiment investigated the effect of joint coil sensitivity map estimation on reconstruction accuracy. XCAT data were simulated for a free‐breathing real‐time scan with simulated sensitivity maps from an 8‐channel array with an acceleration factor of R = 8. Three variants of the Multifrequency Time‐DIP reconstruction were compared, differing in their approach to coil sensitivity estimation: (1) using fixed ground truth sensitivity maps, (2) using fixed ESPIRiT sensitivity maps derived from the time‐averaged k‐space data, and (3) jointly estimating the sensitivity maps during the reconstruction using a CNN to refine the ESPIRiT sensitivity maps.

### 2.7 In Vivo Experiments

Data were collected from 10 healthy subjects and 10 patients undergoing a clinical MRI exam for suspected cardiomyopathy using a 1.5‐T scanner (MAGNETOM Sola, Siemens Healthineers) in an IRB‐approved study after obtaining written informed consent. Four patients exhibited arrhythmias during the exam, including one with PVCs and three with atrial fibrillation. Conventional cine scans were acquired using a 2D breathheld ECG‐gated Cartesian sequence with the following parameters: 1.5 × 1.5 × 8.0 mm^3^, flip angle 60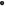, matrix size 224 × 180, FOV 336 × 270 mm^2^, 25 cardiac phases, and breathhold duration of 6–8 s. Real‐time scans without breathholds or ECG gating were acquired using a 2D golden angle spiral bSSFP sequence (2.3 × 2.3 × 8.0 mm^3^, TR = 4.2 ms, flip angle 75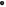). To achieve full LV coverage, 9–12 short‐axis slices were acquired with a 25% (2 mm) slice gap, with each slice requiring 6 s of scan time, resulting in a total of 54–72 s for the entire stack. Cardiac shimming was performed to minimize bSSFP banding artifacts, with the shim volume positioned tightly over the left ventricle.

Real‐time spiral data were binned to a temporal resolution of 25.2 ms (R = 8 with 6 interleaves per frame). To reduce computation demands, data were compressed to 8 virtual coils using the region‐optimized virtual coils (ROViR) method [31]. Images were reconstructed using Multifrequency Time‐DIP with a frequency range of 0.05–20 Hz and joint coil sensitivity estimation. For comparison, reconstructions were also performed using Helix Time‐DIP and CS methods. To demonstrate the feasibility of ultra‐high temporal resolution imaging, real‐time data from a patient with PVCs were reconstructed using only a single spiral interleaf per frame (R = 48, 4.2 ms/frame) using CS, Helix Time‐DIP, and Multifrequency Time‐DIP (frequency range 0.05–120 Hz). Additionally, the impact of joint coil sensitivity estimation was assessed on a separate dataset by reconstructing images with Multifrequency Time‐DIP using (1) fixed ESPIRiT sensitivity maps and (2) joint coil sensitivity estimation (using a CNN to refine the ESPIRiT maps).

To demonstrate the feasibility of imaging during more extreme motion, one healthy subject (30 years, female) was imaged while exercising on a supine ergometer (Lode Medical Technology, Groningen, The Netherlands) inside the scanner bore. Real‐time imaging was performed once the subject reached a target heart rate of 160 bpm, approximately 85% of the age‐predicted maximum (220−age). Spiral data were binned to a temporal resolution of 17 ms/frame (R = 12 with 4 interleaves per frame). A higher temporal resolution was selected here to accommodate the increased motion during exercise. Images were reconstructed using CS, Helix Time‐DIP, and Multifrequency Time‐DIP.

### 2.8 Image Analysis

Image quality was quantitatively evaluated using several metrics. Edge sharpness was assessed by drawing line profiles from the LV myocardium to the LV blood pool on end‐diastolic and end‐systolic frames. Since real‐time scans span multiple heartbeats, only the first complete heartbeat was analyzed. Sharpness was defined as the mean inverse distance between the positions of 20% and 80% maximum intensity along each profile, similar to prior studies [32, 33], with higher values indicating improved sharpness. Twenty profiles were drawn per image, and average sharpness values were reported separately for diastolic and systolic frames at apical, mid, and basal slice positions. Image contrast was measured using the contrast ratio, defined as the ratio between the mean signal intensity in a region of myocardium divided by that in the LV blood pool, with lower values indicating improved contrast. Third, noise levels were quantified using the coefficient of variation (CV), defined as the mean signal intensity divided by the standard deviation within a region of interest (ROI), with CV reported separately for ROIs in myocardium and the LV blood pool. Differences in image quality metrics across scans were analyzed using one‐way ANOVA tests with Tukey's HSD post hoc corrections.

Measurements of LV volumes and function were compared across methods. The LV endocardium was semi‐automatically contoured at end‐diastolic and end‐systolic frames using Medviso Segment [34], with manual corrections applied as needed. End‐diastolic volume (EDV), end‐systolic volume (ESV), and ejection fraction (EF) were measured for conventional cine scans and real‐time scans reconstructed using CS, Helix Time‐DIP, and Multifrequency Time‐DIP methods. Agreement with conventional scans was evaluated using Bland–Altman analyses [35].

Images were presented in a randomized and blinded manner to two board‐certified cardiac imagers with 4 (W.T.) and 8 years (I.R.) of cardiac MRI experience. Each image was evaluated on a 5‐point scale of non‐diagnostic (1), poor (2), average (3), good (4), and excellent (5). The following features were evaluated: sharpness of endocardial borders, blood‐myocardium contrast, temporal dynamics of the LV myocardium, temporal dynamics of the papillary muscles, absence of undersampling artifacts, and apparent SNR. For real‐time scans, the initial one second of data was discarded to avoid transient‐state artifacts. For each rating category, differences in scores across methods were assessed using a Friedman test followed by pairwise Wilcoxon signed‐rank tests with Bonferroni correction.

## 3 Results

### 3.1 Digital Cardiac Phantom Simulations

**FIGURE 3:**
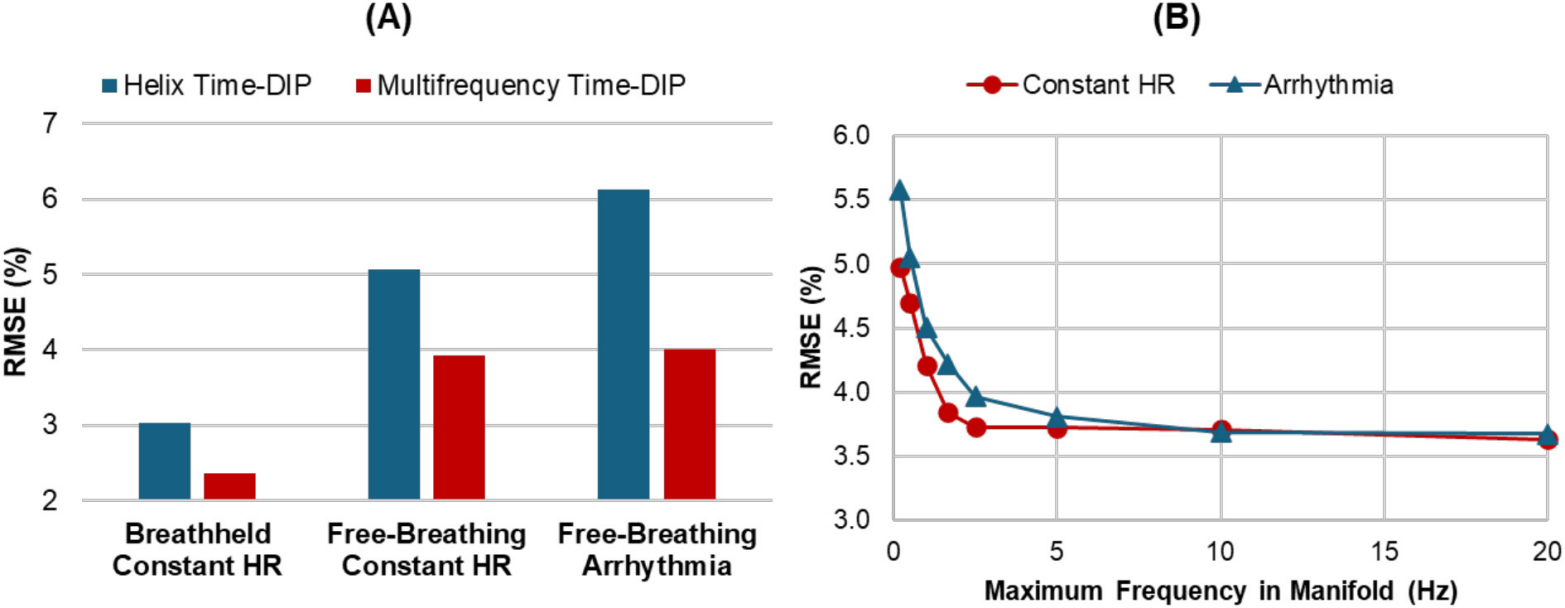
XCAT simulation results. (A) Free‐breathing ungated scans were simulated with an acceleration factor of R = 8 (temporal resolution 25 ms/frame) for three scenarios: (1) a breathheld scan with a constant heart rate (HR) of 70 bpm, (2) a free‐breathing scan with a constant heart rate of 70 bpm, and (3) a free‐breathing scan during a simulated arrhythmia (PVCs). Images were reconstructed using Time‐DIP with a helical manifold (blue) and with the proposed multifrequency manifold (red). (B) RMSE values are shown for Multifrequency Time‐DIP reconstructions performed with varying maximum frequency bounds (the minimum frequency bound was fixed at 0.05 Hz). Free‐breathing ungated scans were simulated for scenarios with (1) a constant heart rate of 70 bpm and (2) an arrhythmia (PVCs).

In simulations with various motion scenarios, both Helix Time‐DIP and Multifrequency Time‐DIP reconstructions exhibited lower errors for breathheld scans compared to free‐breathing scans (Figure 3A). Errors with Helix Time‐DIP progressively increased as the motion became more complex and irregular—from a breathheld scan with constant heart rate (RMSE 3.0%) to a free‐breathing scan with constant heart rate (RMSE 5.1%) and finally to a free‐breathing scan during arrhythmia (RMSE 6.2%). The multifrequency manifold outperformed the helix manifold in all scenarios, particularly during free‐breathing scans. Additionally, the multifrequency manifold resulted in similar errors for free‐breathing scans with a constant heart rate (RMSE 3.9%) and during arrhythmia (RMSE 4.0%).

In general, increasing the maximum frequency bound of the multifrequency manifold (i.e., including higher frequency sinusoids) improved reconstruction accuracy (Figure 3B). For free‐breathing scans having a constant heart rate, increasing the maximum frequency bound reduced the RMSE, with errors plateauing around 2.5 Hz or higher. In contrast, for free‐breathing scans with a simulated arrhythmia, RMSE values continued to decrease as higher frequencies were incorporated into the manifold. In both scenarios, the lowest RMSE was achieved with a maximum frequency bound of 20 Hz, which was the highest value tested (determined by the temporal resolution of the scan).

**FIGURE 4:**
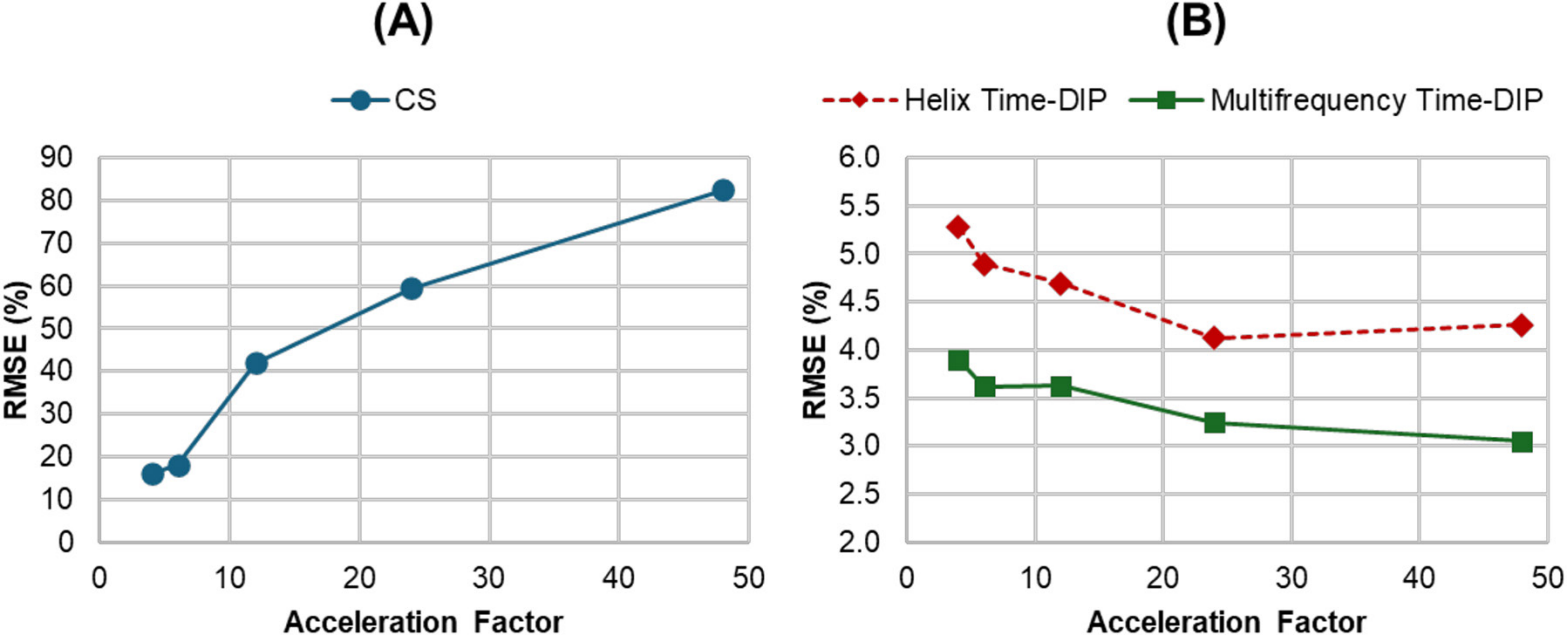
Reconstruction accuracy across different acceleration factors. A free‐breathing ungated golden angle spiral dataset was simulated and different numbers of spiral arms were combined to achieve different temporal resolutions (i.e., different acceleration factors). Data were reconstructed using (A) compressed sensing with temporal total variation regularization (CS), as well as (B) Helix Time‐DIP and Multifrequency Time‐DIP methods. Note the differences in scale on the y‐axes.

Across various acceleration factors, both Helix Time‐DIP and Multifrequency Time‐DIP consistently yielded lower errors than the CS reconstruction, with the multifrequency manifold achieving the lowest RMSE (Figure 4). Interestingly, while CS reconstruction errors increased at higher acceleration factors, both Time‐DIP methods showed a slight decrease in RMSE at higher acceleration rates.

In simulations with multichannel data, joint sensitivity map estimation resulted in lower errors (RMSE 3.6%) compared to using fixed ESPIRiT sensitivity maps derived from a time‐averaged image (RMSE 6.3%), approaching the performance when using the ground truth sensitivity maps (RMSE 3.3%).

### 3.2 In Vivo Experiments

**FIGURE 5:**
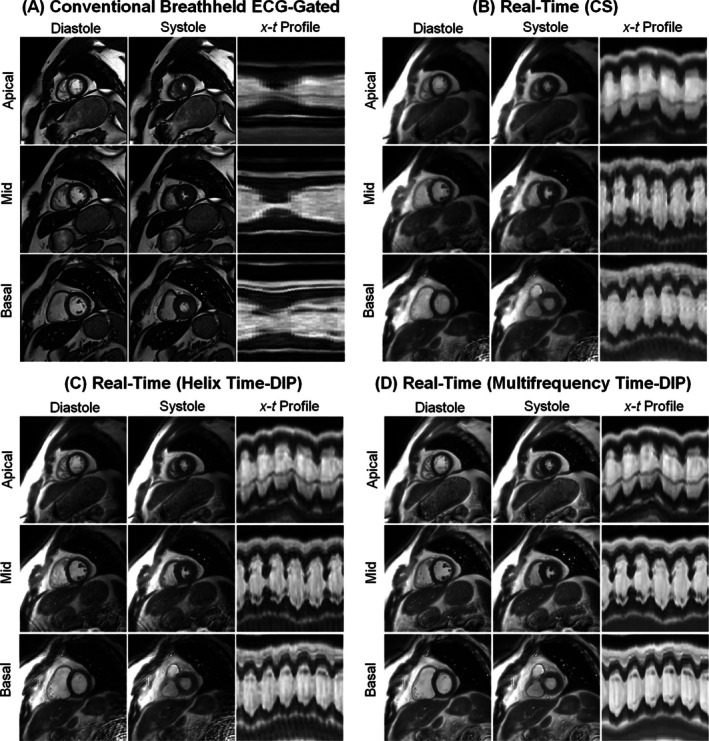
Images from a healthy subject. (A) Conventional cine images are shown, acquired during a 7‐s breathhold using a segmented Cartesian sequence with ECG gating (1.5 × 1.5 × 8.0 mm^3^, 25 phases). Free‐breathing real‐time spiral images acquired in 6‐s per slice are also presented (2.3 × 2.3 × 8.0 mm^3^, 25 ms/frame, R = 8). The same real‐time dataset was reconstructed using (B) CS, (C) Helix Time‐DIP, and (D) Multifrequency Time‐DIP methods. End‐diastolic and end‐systolic frames are displayed, along with a temporal (x‐t) profile passing through the heart at apical, mid, and basal short‐axis slice positions. When examining the temporal profiles, note that the conventional cine represents a single cardiac cycle, while the real‐time scans span several heartbeats.

Examples of conventional Cartesian breathheld ECG‐gated cine images (25 phases) and free‐breathing real‐time spiral images from a healthy subject are presented in Figure 5, where the spiral arms were combined to achieve a temporal resolution of 25 ms/frame (R = 8, or 6 interleaves per frame). The same real‐time dataset was reconstructed using three methods. The CS reconstruction exhibited residual aliasing artifacts, noise enhancement, and motion blurring. While the Helix Time‐DIP reconstruction partially reduced these artifacts, some residual aliasing and motion blurring remained, which was particularly noticeable for fine anatomical features like the papillary muscles (also visible in the x‐t profiles). Multifrequency Time‐DIP with joint sensitivity map estimation yielded the best suppression of aliasing artifacts with minimal blurring. Movies of the conventional cine and real‐time scans are shown in Videos S1 and S2, respectively. In these examples, the average time needed to reconstruct 283 dynamic images acquired during a 6‐s real‐time scan (R = 8) from one slice was 2.7 min for CS, 19.7 min for Helix Time‐DIP, and 20.3 min for Multifrequency Time‐DIP.

**FIGURE 6:**
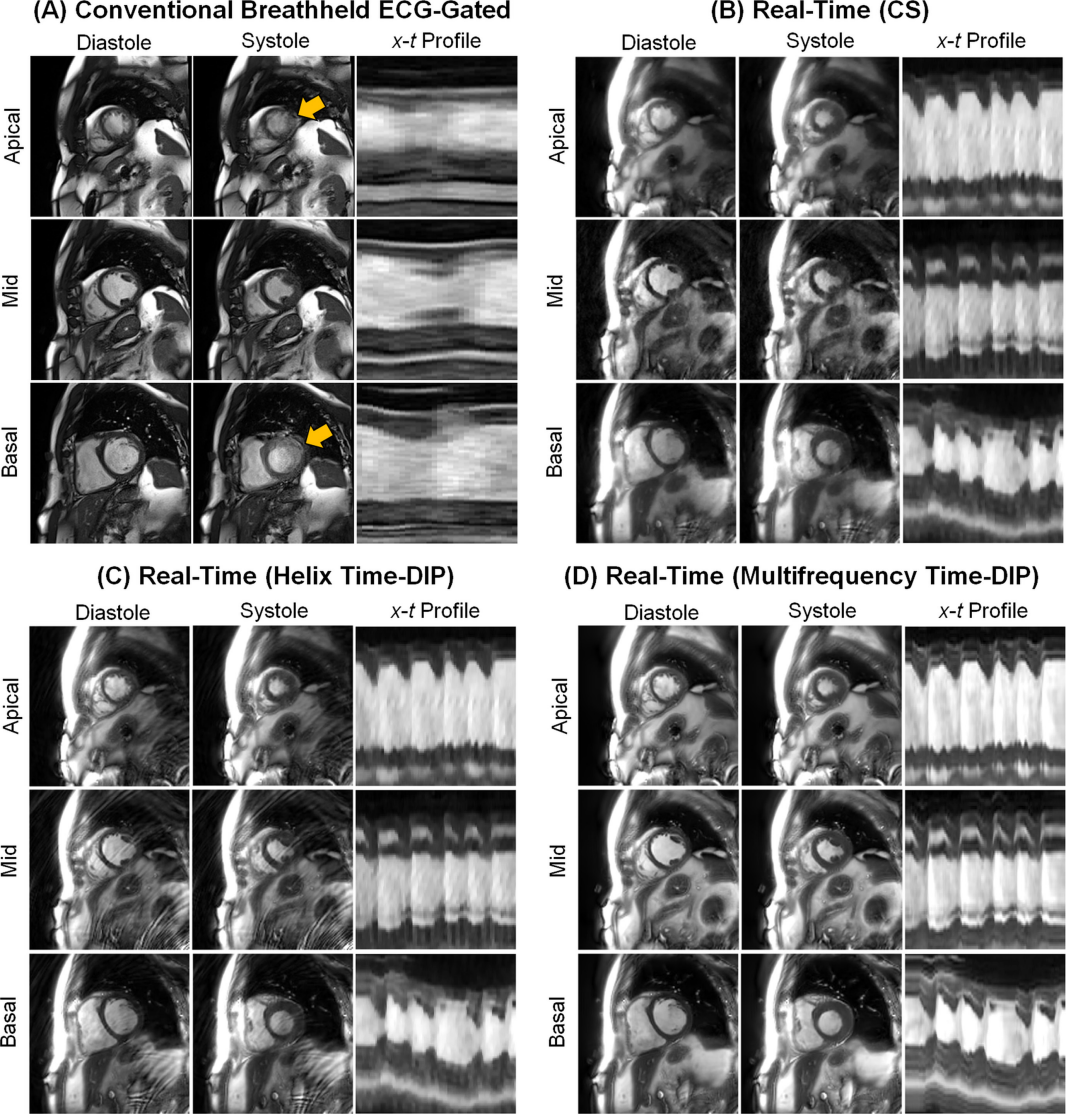
Images from a cardiomyopathy patient experiencing atrial fibrillation during the exam*.* (A) Conventional cine images are shown, acquired during a 7‐s breathhold using a segmented Cartesian sequence with ECG gating (1.5 × 1.5 × 8.0 mm^3^, 25 phases). Motion artifacts from ECG mis‐gating due to the arrhythmia are visible, indicated by yellow arrows. Free‐breathing real‐time spiral images acquired in 6‐s per slice are also presented (2.3 × 2.3 × 8.0 mm^3^, 25 ms/frame, R = 8), which did not exhibit these artifacts as the scans are ungated. The same real‐time dataset was reconstructed using (B) CS, (C) Helix Time‐DIP, and (D) Multifrequency Time‐DIP methods. End‐diastolic and end‐systolic frames are displayed, along with a temporal (x‐t) profile passing through the heart at apical, mid, and basal short‐axis slice positions.

Conventional cine and real‐time free‐breathing images from a patient experiencing atrial fibrillation during the exam are shown in Figure 6. Motion artifacts due to ECG mis‐gating are visible in the conventional scans, particularly in the apical and basal slices. Such artifacts were not observed in the real‐time scans, as they did not employ ECG gating. Irregular cardiac cycle timings can be appreciated in the x‐t profiles for the real‐time scans. In this example, Helix Time‐DIP resulted in prominent spiral aliasing artifacts, likely arising from bright signal from fat, which were largely removed by the Multifrequency Time‐DIP reconstruction with joint sensitivity map estimation. Movies of the conventional cine and real‐time scans from this patient are shown in Videos S3 and S4. Additional images from a patient with PVCs are presented in Videos S5 and S6, where the motion blurring from ECG mis‐gating during the arrhythmia is visible in the conventional cine images but not in the real‐time scans.

**FIGURE 7:**
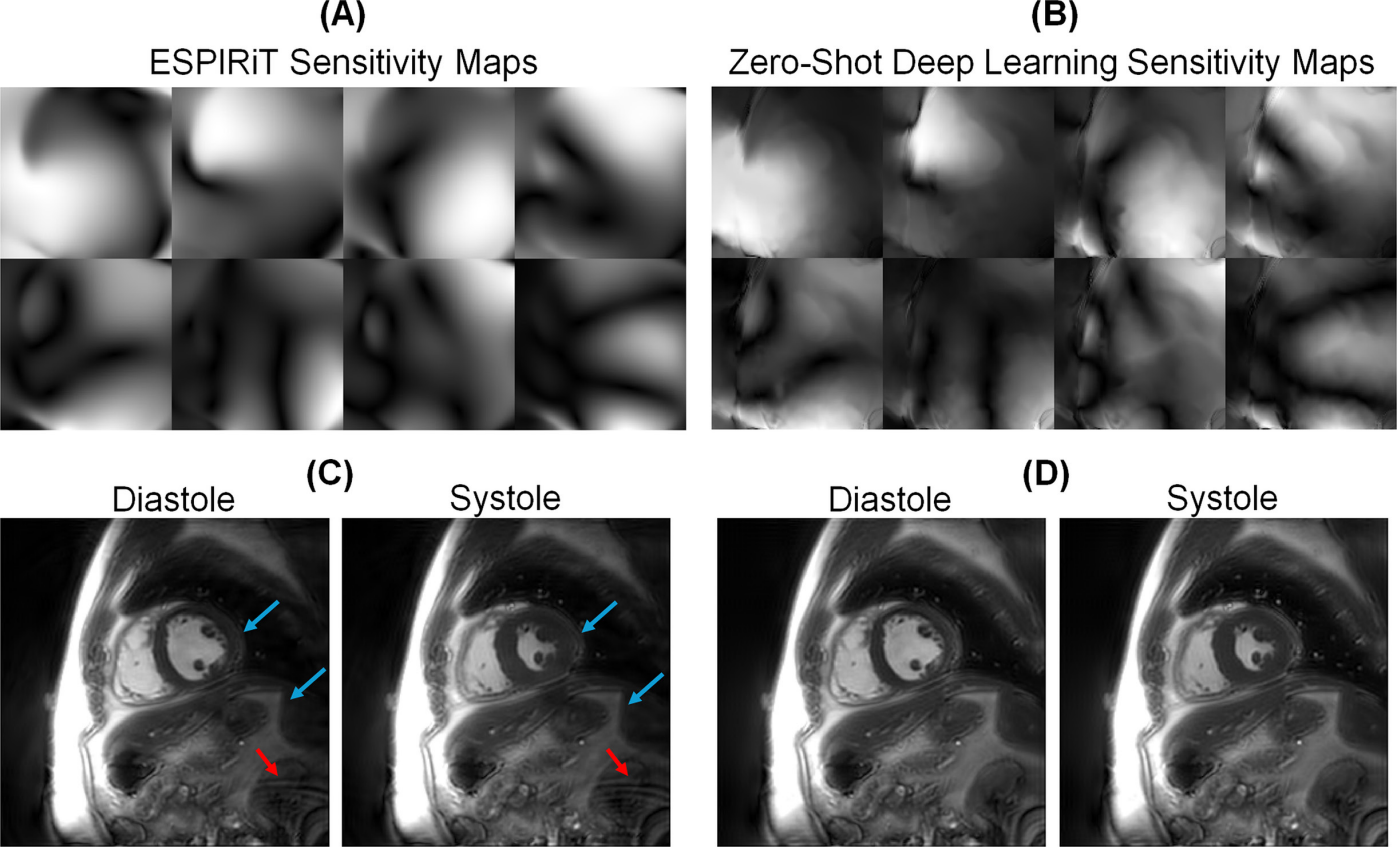
In vivo example demonstrating the effects of joint sensitivity map estimation. (A) ESPIRiT sensitivity maps derived from time‐averaged k‐space data are compared to (B) refined sensitivity maps generated using zero‐shot deep learning. (C) Images in end‐diastole and end‐systole are displayed using Multifrequency Time‐DIP with fixed ESPIRiT sensitivity maps and compared to (D) Multifrequency Time‐DIP with learned sensitivity maps. Red arrows indicate aliasing artifacts that were reduced when using learned sensitivity maps. Joint sensitivity estimation also improved signal homogeneity across the field of view—blue arrows highlight regions with low signal intensity in the reconstruction with fixed sensitivity maps.

As shown in Figure 7, including joint sensitivity estimation in the Multifrequency Time‐DIP reconstruction yielded images with improved signal homogeneity across the field of view (with less shading from receiver coil bias) and reduced aliasing artifacts.

**FIGURE 8:**
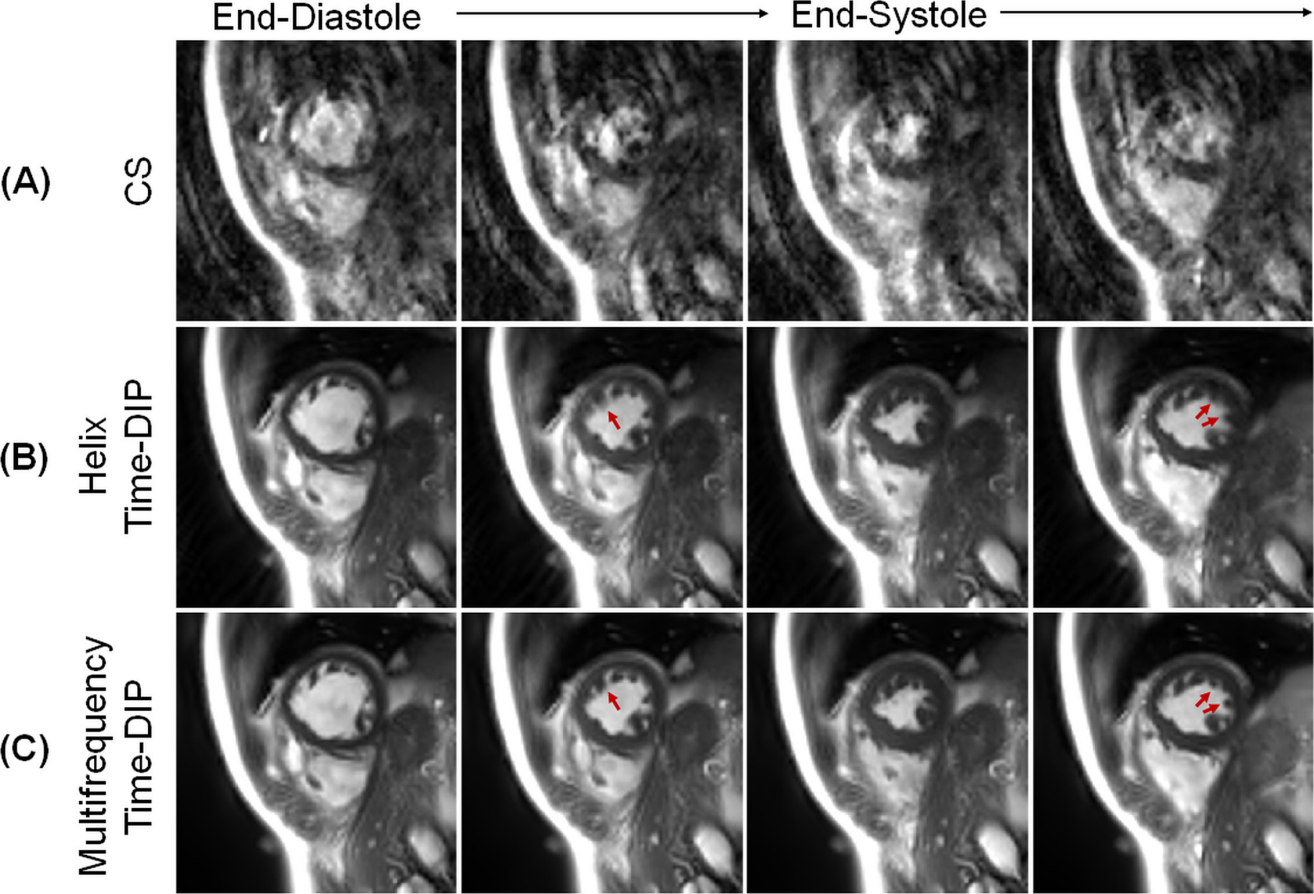
Free‐breathing real‐time images at single‐TR temporal resolution. Real‐time images were reconstructed at a temporal resolution of 4.2 ms per frame (R = 48, one spiral interleaf binned per frame) using (A) CS, (B) Helix Time‐DIP, and (C) Multifrequency Time‐DIP methods. Four representative frames are displayed—the first and third columns show end‐diastolic and end‐systolic frames, while the second and fourth columns depict intermediate cardiac phases. Red arrows indicate slight motion blurring in the papillary muscles on Helix Time‐DIP reconstructions, which appear sharper with Multifrequency Time‐DIP.

Real‐time images from a patient with PVCs reconstructed at a single‐TR temporal resolution of 4.2 ms per frame (R = 48) are presented in Figure 8. CS images had severe aliasing artifacts, whereas image quality substantially improved with both Helix Time‐DIP and Multifrequency Time‐DIP reconstructions. Helix Time‐DIP resulted in increased residual aliasing (evident in Video S7) and motion blurring in fine anatomical structures like the papillary muscles compared to Multifrequency Time‐DIP. Compared to the R = 8 reconstructions described above, the R = 48 datasets required longer reconstruction times (approximately 10 min for CS and 2 h for both Helix and Multifrequency Time‐DIP) due to the larger number of images (1429 frames) acquired during the 6‐s scan.

**FIGURE 9:**
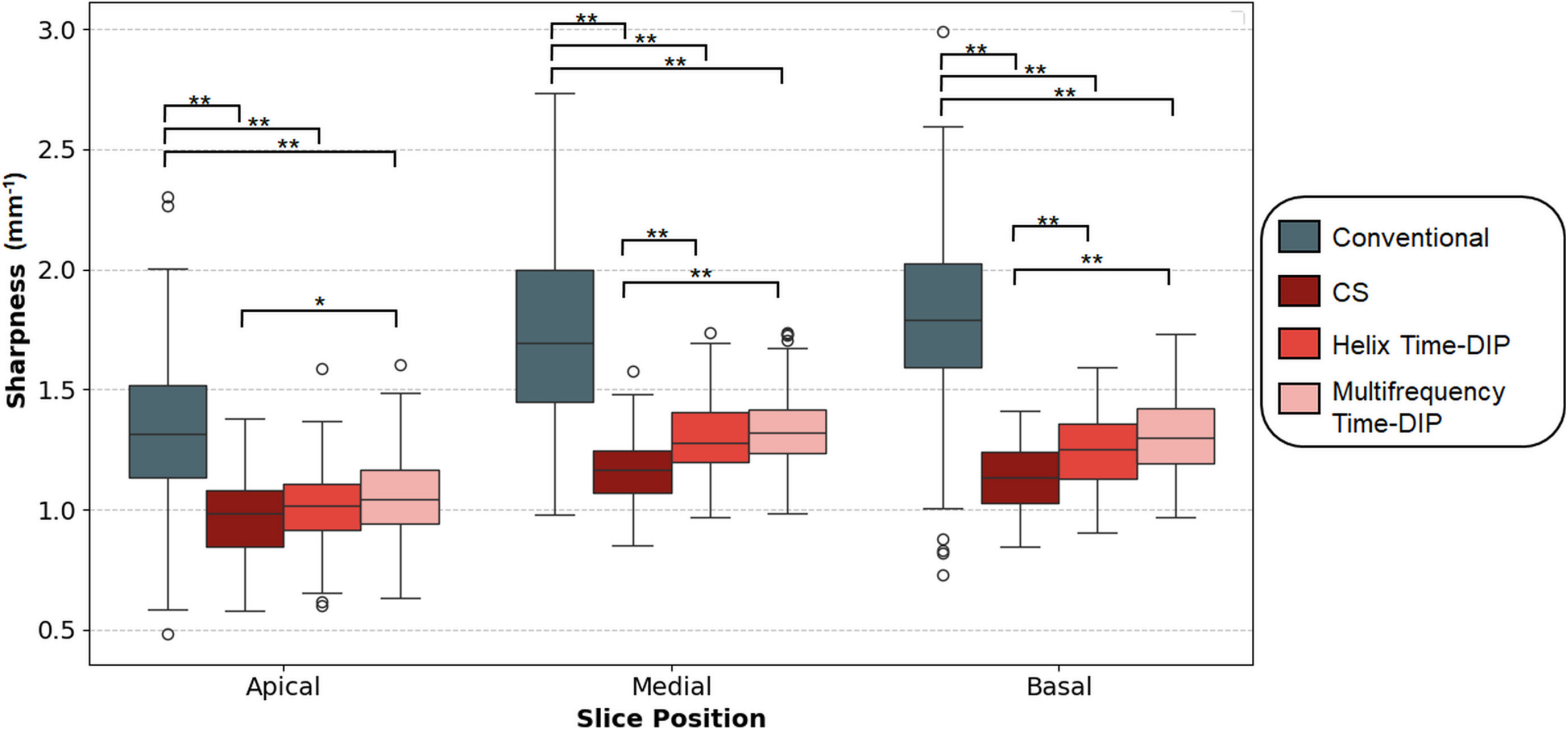
Quantitative image sharpness comparison. Conventional breathheld and ECG‐gated Cartesian cine images (blue) exhibited the highest (best) sharpness scores across apical, mid, and basal slice positions. Among free‐breathing ungated images, Multifrequency Time‐DIP achieved the highest sharpness scores, followed by Helix Time‐DIP and CS reconstructions. Asterisks denote statistical significance, with (*) indicating 0.01 ≤ *p* < 0.05 and (**) indicating *p* < 0.01.

**FIGURE 10:**
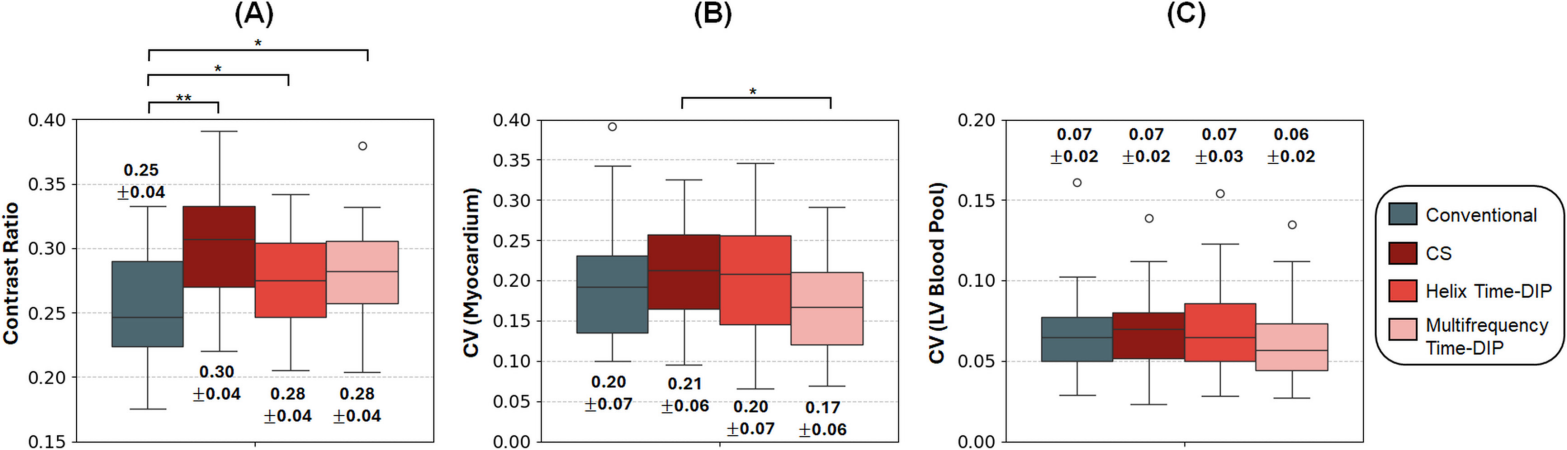
Quantitative contrast and noise image quality metrics. (A) The contrast ratio is presented, defined as the signal intensity ratio of myocardium relative to LV blood. Conventional breathheld ECG‐gated Cartesian cine scans exhibited the best (lowest) contrast ratios. Among real‐time scans, Helix Time‐DIP and Multifrequency Time‐DIP demonstrated slightly better contrast ratios on average compared to CS. (B) Coefficient of variation (CV) measurements are shown for regions of interest in myocardium and (C) the LV blood pool. Lower CV values indicate improved signal homogeneity and reduced noise. Multifrequency Time‐DIP produced slightly lower CV values in both myocardium and blood compared to other methods, including conventional cine scans. Asterisks denote statistical significance, with (*) indicating 0.01 ≤ *p* < 0.05 and (**) indicating *p* < 0.01.

Figures 9 and 10 compare image quality metrics between real‐time and conventional breathheld ECG‐gated Cartesian cine images. Edge sharpness was significantly lower for all real‐time scans compared to conventional images; however, it should be noted that conventional scans were acquired at higher in‐plane resolution (1.5 × 1.5 mm^3^ versus 2.3 × 2.3 mm^3^) with breathholding and ECG gating. Among real‐time methods, Multifrequency Time‐DIP achieved the best edge sharpness metrics for all slice locations (apical, mid, and basal), with significantly improved results compared to the CS reconstruction. Conventional cine scans exhibited the highest contrast between blood and myocardium (i.e., lowest contrast ratio scores). However, among real‐time images, Multifrequency and Helix Time‐DIP resulted in improved (lower contrast ratio) scores compared to the CS reconstruction. CV measurements within both myocardium and blood regions were lowest for real‐time Multifrequency Time‐DIP images compared all other methods, although these differences were not statistically significant.

**FIGURE 11:**
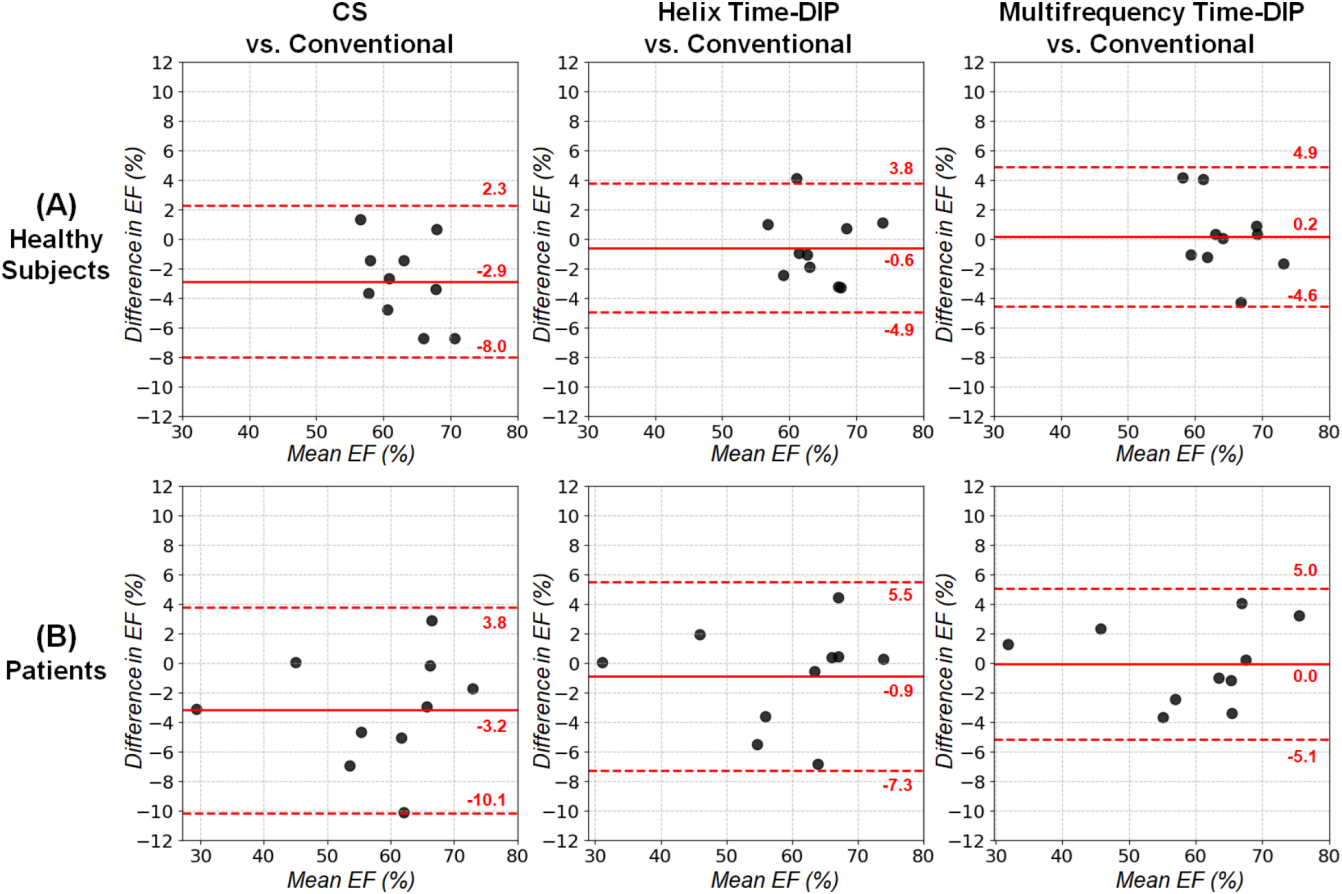
Bland–Altman plots for left ventricular EF. Results are shown in (A) healthy subjects and (B) cardiomyopathy patients comparing EF from real‐time spiral scans (R = 8, 25 ms/frame) reconstructed with CS, Helix Time‐DIP, and Multifrequency Time‐DIP methods against measurements from conventional breathheld ECG‐gated Cartesian cine scans. Each plot displays the mean bias (solid red line) and 95% limits of agreement (dotted red lines).

**FIGURE 12:**
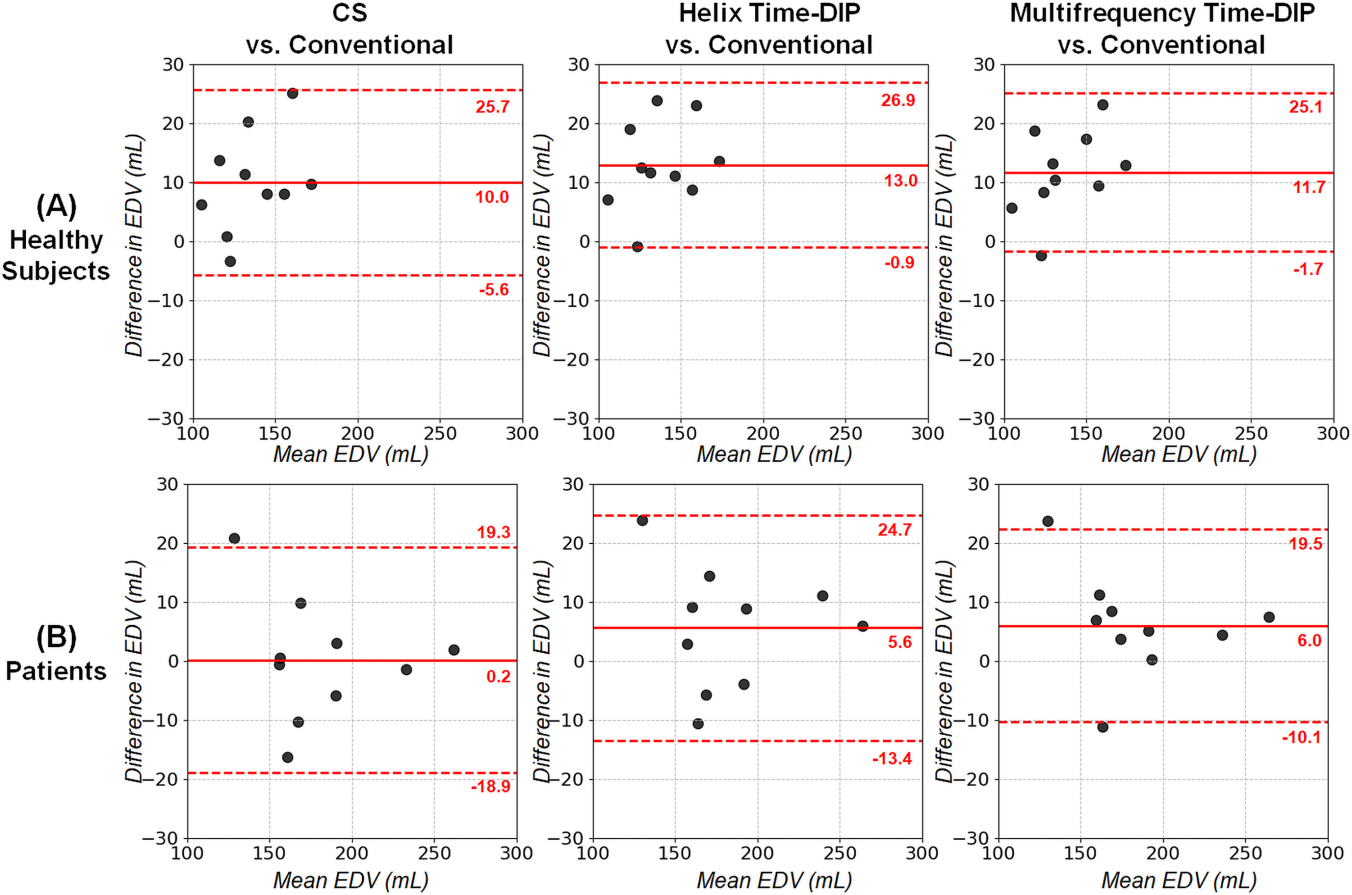
Bland–Altman plots for left ventricular EDV. Results are shown in (A) healthy subjects and (B) cardiomyopathy patients comparing EDV from real‐time spiral scans (R = 8, 25 ms/frame) reconstructed with CS, Helix Time‐DIP, and Multifrequency Time‐DIP methods against measurements from conventional breathheld ECG‐gated Cartesian cine scans. Each plot displays the mean bias (solid red line) and 95% limits of agreement (dotted red lines).

**FIGURE 13:**
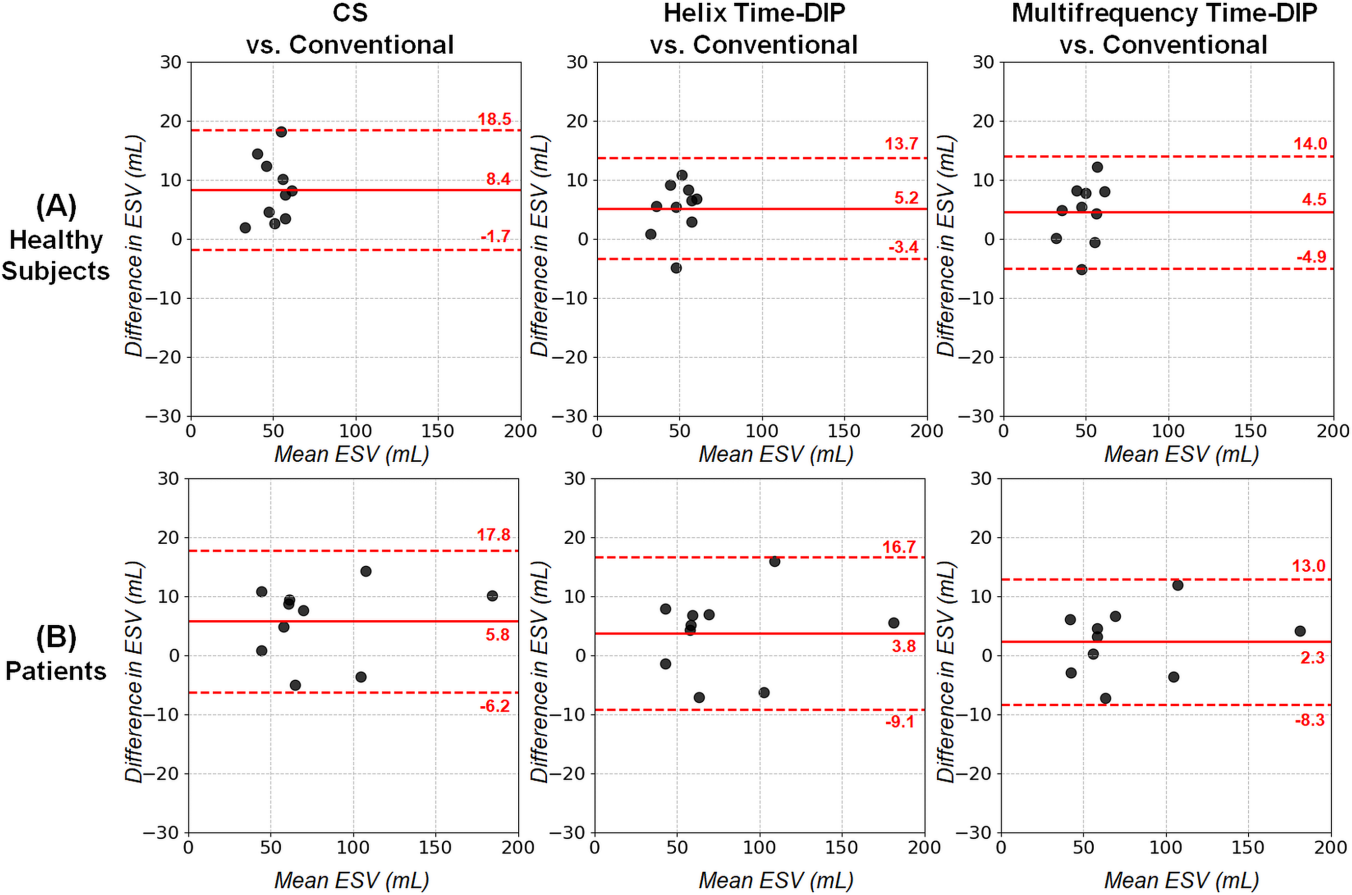
Bland–Altman plots for left ventricular ESV. Results are shown in (A) healthy subjects and (B) cardiomyopathy patients, comparing ESV from real‐time spiral scans (R = 8, 25 ms/frame) reconstructed with CS, Helix Time‐DIP, and Multifrequency Time‐DIP methods against measurements from conventional breathheld ECG‐gated Cartesian cine scans. Each plot displays the mean bias (solid red line) and 95% limits of agreement (dotted red lines).

With all real‐time methods, no significant differences in EF were observed relative to conventional scans in Bland–Altman analyses (Figure 11). The mean bias in EF values with Multifrequency Time‐DIP was 0.2% in healthy subjects and 0.0% in patients. Among real‐time methods, Multifrequency Time‐DIP yielded narrower 95% limits of agreement (−5.1% to 5.0%) in patients compared to Helix Time‐DIP (−7.3% to 5.5%) and CS (−10.1% to 3.8%). Similarly, no significant differences were seen with any real‐time method relative to conventional scans for EDV (Figure 12) and ESV (Figure 13). There was a non‐significant trend towards slightly higher EDV and ESV measurements in healthy subjects with real‐time imaging, regardless of reconstruction method, compared to conventional scans.

**FIGURE 14:**
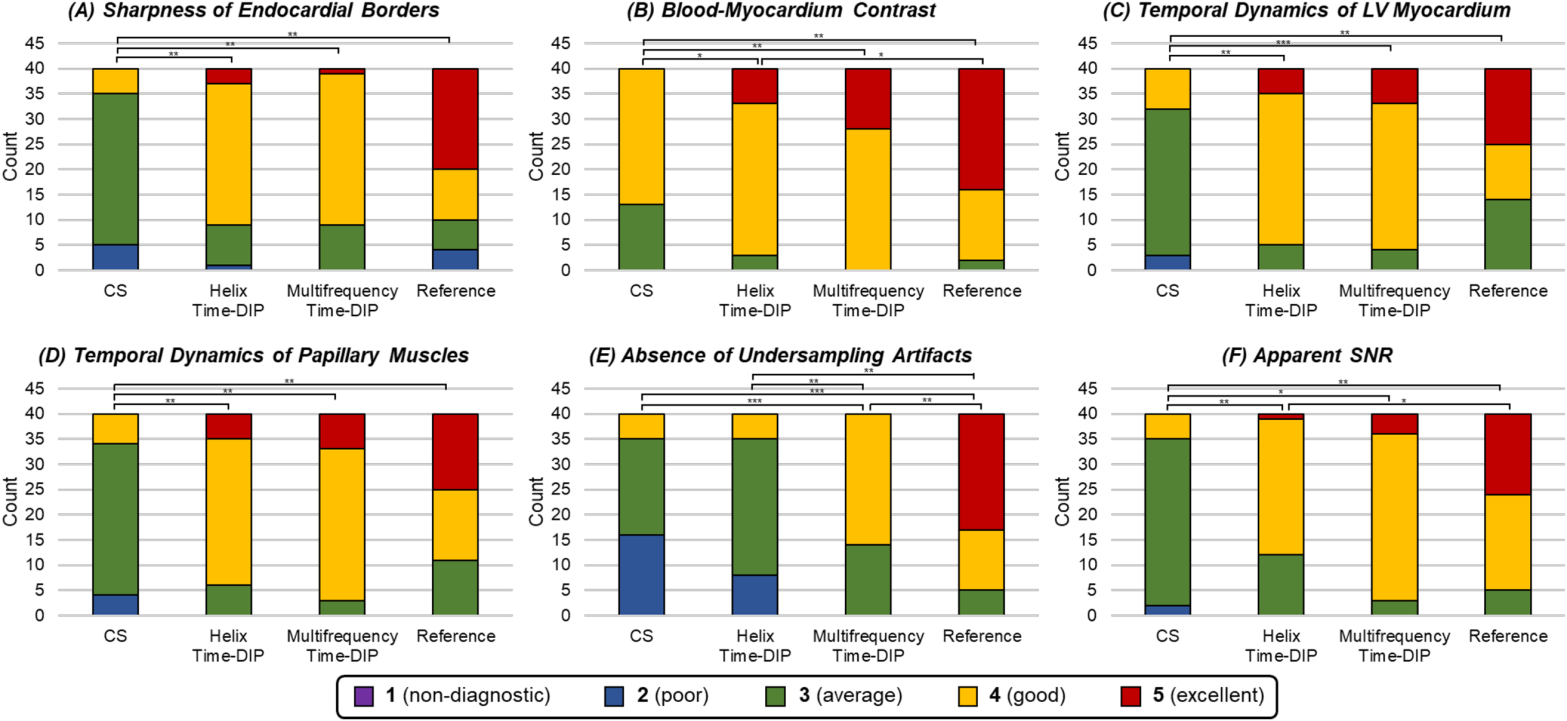
Summary of image quality ratings. The distribution of scores pooled from both reviewers is shown for each of the six image feature categories: (A) sharpness of endocardial borders, (B) blood‐myocardium contrast, (C) temporal dynamics of LV myocardium, (D) temporal dynamics of papillary muscles, (E) absence of undersampling artifacts, and (F) apparent SNR. Asterisks denote statistical significance (* for *p* < 0.05, ** for *p* < 0.01, *** for *p* < 0.001).

Real‐time CS images were rated significantly lower than both Time‐DIP reconstructions (with helix and multifrequency manifolds) and reference cine images across all categories (Figure 14). Helix Time‐DIP received significantly lower ratings than reference images for blood‐myocardium contrast, undersampling artifacts, and apparent SNR. In contrast, ratings for real‐time Multifrequency Time‐DIP did not differ significantly from reference images in five of the six categories. The only exception was the undersampling artifacts category, where reference images were rated as having significantly fewer artifacts compared to all real‐time methods; however, Multifrequency Time‐DIP outperformed both CS and Helix Time‐DIP in this category.

**FIGURE 15:**
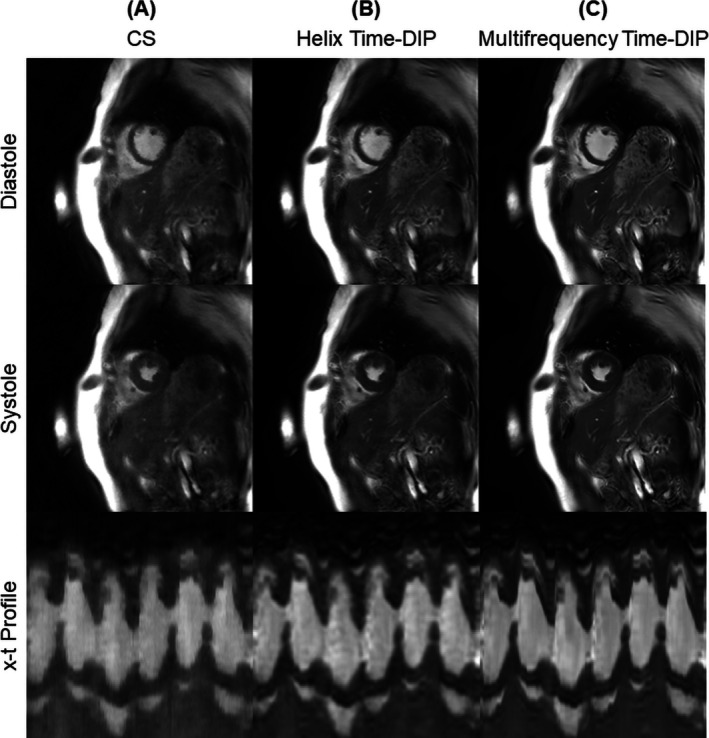
Real‐time imaging during in‐bore exercise stress. Real‐time images were acquired from a healthy subject exercising on a supine ergometer inside the scanner bore. Data were reconstructed at a temporal resolution of 16.8 ms (R = 12, 4 spiral interleaves binned per frame) using (A) CS, (B) Helix Time‐DIP, and (C) Multifrequency Time‐DIP. Representative frames are displayed at end‐diastole and end‐systole, along with a temporal (x‐t) profile through the heart.

The proposed method was demonstrated to be feasible for real‐time exercise stress imaging at high temporal resolution (R = 12, 17 ms/frame), as shown in Figure 15. Multifrequency Time‐DIP yielded fewer aliasing artifacts and reduced motion blurring compared to CS and Helix Time‐DIP reconstructions of the same data, despite the presence of substantial bulk movement from bicycle pedaling (Video S8).

## 4 Discussion

This study extends the Time‐Dependent Deep Image Prior framework to free‐breathing real‐time cardiac imaging by introducing a novel multifrequency manifold, along with joint coil sensitivity estimation to improve the reconstruction of multichannel data. The proposed Multifrequency Time‐DIP reconstruction was evaluated in healthy subjects and patients, including those with arrhythmias, achieving temporal resolutions as high as single TR (4.2 ms/frame). Real‐time golden angle spiral bSSFP imaging with Multifrequency Time‐DIP demonstrated superior suppression of aliasing artifacts and improved image quality metrics compared to the original Time‐DIP reconstruction (referred to here as “Helix Time‐DIP”), which employed a helical manifold and fixed sensitivity maps, and compressed sensing with temporal total variation regularization. All real‐time methods, including Multifrequency Time‐DIP, produced LV functional measurements within clinically acceptable ranges compared to conventional cine scans (e.g., mean bias < 5% for LVEF), with Multifrequency Time‐DIP demonstrating slightly narrower limits of agreement in most cases compared to other methods.

Free‐breathing real‐time imaging is especially beneficial for patients with limited breathhold capacity or arrhythmias. Conventional cine scans use segmented acquisitions with retrospective ECG gating, where data collected over multiple heartbeats are reordered based on the time after the R‐wave in the ECG signal, yielding images that depict motion over a single “average” cardiac cycle. These images are prone to artifacts if there is excessive respiratory motion or during arrhythmias, which hampers reliable ECG gating, resulting in the combination of inconsistent data across heartbeats. In this study, four of 10 patients exhibited arrhythmias during the exam, including atrial fibrillation and PVCs. In two cases (Figure 6 and Videos S3–S6), ECG mis‐gating resulted in motion artifacts in the conventional scans. The proposed method, which did not employ ECG gating, was free of these artifacts and furthermore enabled real‐time visualization of cardiac motion during the arrhythmia.

Through‐plane motion, such as respiratory displacement, may cause focal abnormalities to move in and out of the imaging plane in 2D real‐time scans, or cause inaccuracies in volume and EF measurements due to slice misregistration artifacts. However, this is a limitation of 2D cine imaging in general and is not specific to our method. Moreover, while respiration can cause through‐plane motion in free‐breathing real‐time scans, slice misregistration also affects conventional breath‐held cine scans, particularly if subjects cannot maintain consistent breath‐hold positions across multiple slices.

Significantly improved edge sharpness was observed in the conventional cine scans compared to the real‐time scans, regardless of reconstruction method. This is likely due to several factors: (1) unlike the real‐time scans, conventional scans employed breath‐holding and ECG gating, (2) conventional scans were acquired at a higher in‐plane spatial resolution (1.5 × 1.5 mm^2^ vs. 2.3 × 2.3 mm^2^), and (3) no correction for spiral off‐resonance blurring was performed for the real‐time scans, although the spiral readout was quite short (2.4 ms).

A key innovation of this work is the multifrequency manifold design, which encodes temporal variations using sinusoids spanning a broad range of frequencies. Lower frequency sinusoids enable the recovery of slower dynamics (e.g., respiration), while higher frequency sinusoids facilitate recovery of faster dynamics (e.g., cardiac motion). The multifrequency manifold is conceptually similar to the idea of positional encoding in transformer networks, where sinusoidal functions embed positional information within a latent space [36]. Here, instead of encoding spatial information, the proposed approach encodes temporal position by sampling a set of sinusoids covering a range of frequencies, ensuring that each time frame is represented by a unique set of values. Unlike the helical manifold used in the original Time‐DIP approach, this method eliminates the need for scan‐specific tuning of the manifold and makes no assumptions about motion periodicity, making it suitable for free‐breathing scans and real‐time imaging during arrhythmias.

The frequency range used to construct the multifrequency manifold was found to be a critical parameter affecting the reconstruction accuracy. Incorporating higher frequency sinusoids led to improved reconstruction accuracy across various simulated motion scenarios, including breathheld and free‐breathing scans, particularly for arrhythmias. Based on these results, the maximum frequency bound for all subsequent in vivo reconstructions was set to $\frac{1}{2T}$ Hz, where T was the temporal resolution (in seconds) of the scan, to allow the manifold to capture the highest temporal variations resolvable by the scan.

Another contribution of this work is the integration of coil sensitivity estimation within the zero‐shot deep learning framework. This approach reduced errors in simulations compared to using pre‐calculated ESPIRiT sensitivity maps derived from the time‐averaged k‐space data. In vivo reconstructions exhibited improved signal homogeneity and reduced residual aliasing with joint coil sensitivity estimation.

Multifrequency Time‐DIP employs zero‐shot learning, where the image reconstruction and coil sensitivity CNNs are trained de novo using undersampled k‐space data from a single scan, avoiding the need for fully‐sampled training data. This approach is particularly advantageous for real‐time cardiac imaging applications, where acquiring fully‐sampled training datasets is often infeasible due to respiratory and cardiac motion. A well‐known challenge with zero‐shot learning techniques, such as the Deep Image Prior, is determining the optimal number of training epochs to prevent overfitting to noise or artifacts due to the lack of ground truth training data [16]. This study addressed this issue by training with dropout regularization [24], as in previously described DIP techniques for real‐time imaging [37] and MR Fingerprinting [38]. As shown in Figure S5, dropout reduced reconstruction errors and stabilized training. Without dropout, errors initially decreased but then rapidly increased due to overfitting, requiring precise selection of the number of epochs to achieve the best reconstruction. In contrast, training with dropout caused the errors to decrease to a more stable plateau, reducing sensitivity to the number of training epochs and leading to lower overall errors.

There are stochastic elements in the Time‐DIP reconstruction, including the random selection of time frames in mini‐batches, random initialization of network weights, and use of a stochastic optimization algorithm (Adam). Thus, repeated reconstructions of the same dataset may yield slightly different outcomes. However, in practice, this variability was observed to be small. In simulations of a free‐breathing scan during arrhythmia (Figure S6), the mean and standard deviation of RMSE values over 10 repeated reconstructions were 4.0% ± 0.1% for Multifrequency Time‐DIP and 6.2% ± 0.1% for Helix Time‐DIP.

Multifrequency Time‐DIP has some similarities with other zero‐shot deep learning methods used for real‐time cardiac imaging. The DEBLUR [39] and low‐rank deep image prior (LR‐DIP) [37] techniques both employ CNNs with zero‐shot training to generate spatial and temporal basis functions, which are multiplied to produce dynamic images. However, a key distinction is that these methods represent real‐time images using a linear low‐rank subspace model, whereas Multifrequency Time‐DIP employs a nonlinear manifold. The deep Generative SmooThness Regularization on Manifolds (Gen‐SToRM) technique combines a CNN (with zero‐shot training) with a learned manifold for image reconstruction [17]. However, Gen‐STORM derives an initial estimate of the manifold from low frequency k‐space data and then updates both the CNN and manifold during training, requiring several tunable hyperparameters. In contrast, Multifrequency Time‐DIP uses a fixed manifold designed to be general enough to encode various types of motion, with the frequency bounds serving as the main tunable hyperparameter. Another recent zero‐shot approach is the Motion‐Guided Deep Image Prior (M‐DIP), which captures both motion and contrast variations by non‐rigidly warping a time‐varying template image derived from a spatial dictionary [40]. In contrast to Multifrequency Time‐DIP, which encodes temporal dynamics implicitly using a pre‐defined manifold, M‐DIP models motion explicitly using deformation fields. Both the template image and deformation fields are generated by networks employing zero‐shot training, which are jointly optimized during the reconstruction.

This study has several limitations and opportunities for future work. First, the current computation time poses a barrier to clinical deployment. Because network training is performed de novo for each scan, it takes approximately 20 min to reconstruct 283 real‐time image frames (6‐s scan duration) at R = 8 from one slice on a single GPU, while reconstructing 1429 image frames at R = 48 (also a 6‐s scan duration) requires 2 h. Future work will explore using parallel GPUs or transfer learning to reduce computation time, wherein networks could be pre‐trained on existing datasets and then fine‐tuned for individual scans. Second, while this study demonstrated that a single‐TR temporal resolution is feasible, such high frame rates are not typically necessary for assessing LV function. Future work will explore applications where ultra‐high temporal resolution is advantageous, such as real‐time imaging of cardiac valves, exercise stress imaging, and pediatric applications [41]. Third, compared to conventional cine scans (1.5 × 1.5 mm^2^), real‐time images were acquired at a lower in‐plane spatial resolution (2.3 × 2.3 mm^2^) to ensure sufficient SNR and image quality under high undersampling conditions (up to R = 48). Future studies will investigate real‐time imaging at a higher spatial resolution, which would be beneficial for various applications, including assessment of right ventricular function. Fourth, the sample size of this study was small, comprising 10 healthy subjects and 10 patients, of whom only 4 exhibited arrhythmias. Thus, this work was not powered for statistical comparisons within the subgroup of arrhythmia patients. Additional prospective validation in larger and diverse patient cohorts is required, particularly those with arrhythmias, and in multi‐site and multi‐vendor settings to assess generalizability. Finally, this technique could be extended to other time‐resolved MRI applications, including myocardial first‐pass perfusion and parametric mapping using methods like MR Fingerprinting [42].

## Conflicts of Interest

Jesse Hamilton, Gastao Cruz, and Nicole Seiberlich receive research grant support from Siemens Healthineers.

## Supporting information


**Figure S1:** Schematic of the original Time‐DIP reconstruction.
**Figure S2:** Effect of the height, width, and channels of the input to the Image Reconstruction CNN. RMSE values are shown for (A) various height and width dimensions and (B) different numbers of network channels for the input $z_{t}$ to the CNN.
**Table S1:** Simulations comparing Helix and Multifrequency Time‐DIP reconstructions both with and without MapNet.
**Figure S3:** Architecture of the Image Reconstruction CNN. **(A)** The multifrequency manifold is sampled for a specific time frame t. The manifold sample $z_{t}$, which has a size of H×W×C (height × width × channels), is processed by a CNN consisting of several blocks. **(B)** Each block includes 3 × 3 convolutions, ReLU activations, dropout, and 2 × 2 nearest neighbor interpolation (upsampling). The number of blocks in the CNN depends on the height and width of $z_{t}$ and of the reconstructed image $x_{t}$. This study required four blocks, since four upsampling steps were needed to given the dimensions of the input $z_{t}$ (height and width of 8 × 8) and the final image (128 × 128). **(C)** The last block in the CNN differs slightly from the rest, as it lacks a final activation function and does not perform upsampling. **(D)** The CNN outputs a complex‐valued image, with two channels for the real and imaginary components.
**Figure S4:** Architecture of the CNN for coil sensitivity estimation. **(A)** An initial estimate of the sensitivity maps is derived from time‐averaged data using ESPIRiT. These maps are reshaped into a tensor of size $N_{y}\times N_{x}\times 2N_{c}$, where $N_{c}$ represents the number of MRI receiver coils and the factor of 2 accounts for the real and imaginary components. **(B)** The CNN consists of four blocks, each containing a dropout layer, 3 × 3 convolution, and ReLU activation function. **(C)** The final output is a refined estimate of the coil sensitivity maps.
**Figure S5:** Simulation results showing RMSE versus training epochs with different levels of dropout regularization.
**Figure S6:** Simulation results showing the distribution of RMSE values across 10 repeated reconstructions of the same dataset using Helix Time‐DIP and Multifrequency Time‐DIP. Asterisks indicate statistical significance (*p* < 0.001).

**Video S1:** Real‐time spiral images (R = 8, 25 ms/frame) from a healthy subject are shown at apical, mid, and basal slice positions. From left to right, each column shows the same dataset reconstructed using CS (left), Helix Time‐DIP (middle), and Multifrequency Time‐DIP (right) methods. This movie corresponds to the same dataset shown in Figure 5.

**Video S2:** Conventional breathheld ECG‐gated cine images are presented from the same healthy subject as in Figure 5 and Video S1.

**Video S3:** Real‐time spiral images (R = 8, 25 ms/frame) from a patient with atrial fibrillation are shown at apical, mid, and basal slice positions. From left to right, each column shows the same dataset reconstructed using CS (left), Helix Time‐DIP (middle), and Multifrequency Time‐DIP (right) methods. This movie corresponds to the same dataset shown in Figure 6.

**Video S4:** Conventional breathheld ECG‐gated cine images are presented from the same patient with atrial fibrillation shown in Figure 6 and Video S3. ECG mis‐gating artifacts and blurring are visible particularly in the apical and basal slices. These artifacts are not seen on the real‐time scans in Video S3.

**Video S5:** Real‐time spiral images (R = 8, 25 ms/frame) from a patient with premature ventricular contractions are shown at apical, mid, and basal slice positions. From left to right, each column shows the same dataset reconstructed using CS (left), Helix Time‐DIP (middle), and Multifrequency Time‐DIP (right) methods.

**Video S6:** Conventional breathheld ECG‐gated cine images are presented from the same patient with premature ventricular contractions as in Video S5.

**Video S7:** Real‐time spiral images from a patient with premature ventricular contractions are shown at a temporal resolution equal to a single TR (4.2 ms/frame, one spiral interleaf per frame, R = 48). Images were reconstructed using CS (left), Helix Time‐DIP (middle), and Multifrequency Time‐DIP (right) methods.

**Video S8:** Real‐time stress images at peak exercise are shown from a healthy subject pedaling inside the scanner on a supine ergometer (still images are shown in Figure 15). The real‐time spiral data were binned to a temporal resolution of 17 ms/frame (R = 12, 4 spiral interleaves per frame) and reconstructed using CS (left), Helix Time‐DIP (middle), and Multifrequency Time‐DIP (right) methods.

## Data Availability

The data that support the findings of this study are available from the corresponding author upon reasonable request.
