# Supplementary material for: Multifrequency Time‐Dependent Deep Image Prior for Real‐Time Free‐Breathing Cardiac Imaging
Source: NMR Biomed. 2025 Aug 4;38(9):e70114. doi: 10.1002/nbm.70114 (PMC12322519; doi:10.1002/nbm.70114)
Supplement: Supplementary file 1 — Figure S1: Schematic of the original Time‐DIP reconstruction. Figure S2: Effect of the height, width, and channels of the input to the Image Reconstruction CNN. RMSE values are shown for (A) various height and width dimensions and (B) different numbers of network channels for the input zt to the CNN. Table S1: Simulations comparing Helix and Multifrequency Time‐DIP reconstructions both with and without MapNet. Figure S3: Architecture of the Image Reconstruction CNN. (A) The multifrequency manifold is sampled for a specific time frame t. The manifold sample zt, which has a size of H×W×C (height × width × channels), is processed by a CNN consisting of several blocks. (B) Each block includes 3 × 3 convolutions, ReLU activations, dropout, and 2 × 2 nearest neighbor interpolation (upsampling). The number of blocks in the CNN depends on the height and width of zt and of the reconstructed image xt. This study required four blocks, since four upsampling steps were needed to given the dimensions of the input zt (height and width of 8 × 8) and the final image (128 × 128). (C) The last block in the CNN differs slightly from the rest, as it lacks a final activation function and does not perform upsampling. (D) The CNN outputs a complex‐valued image, with two channels for the real and imaginary components. Figure S4: Architecture of the CNN for coil sensitivity estimation. (A) An initial estimate of the sensitivity maps is derived from time‐averaged data using ESPIRiT. These maps are reshaped into a tensor of size Ny×Nx×2Nc, where Nc represents the number of MRI receiver coils and the factor of 2 accounts for the real and imaginary components. (B) The CNN consists of four blocks, each containing a dropout layer, 3 × 3 convolution, and ReLU activation function. (C) The final output is a refined estimate of the coil sensitivity maps. Figure S5: Simulation results showing RMSE versus training epochs with different levels of dropout regularization. Figure S6: Simulation results show [file NBM-38-e70114-s003.docx]

**Supporting Information**

# *1. Overview of the Time-Dependent Deep Image Prior*

**Supporting Figure S1** illustrates an overview of the original Time-DIP reconstruction (Yoo J, Jin KH, Gupta H, Yerly J, Stuber M, Unser M. Time-Dependent Deep Image Prior for Dynamic MRI. IEEE Trans Med Imaging. 2021;40(12):3337-3348). The temporal dimension of a dynamic scan is parameterized using a low-dimensional manifold. For a specific time frame index $t$, this manifold is sampled to produce a vector $z_{t}$. The original study tested various manifold designs and found that a three-dimensional helical manifold provided the best performance for breathheld real-time (ungated) cardiac imaging. The vector $z_{t}$ is then input into a fully connected network $g$ (also referred to as “MapNet” by Yoo et al.), which transforms the manifold to a more expressive latent space, utilizing two hidden layers with 300 nodes each. The output of MapNet is a 64-element vector, which is reshaped to an 8x8 matrix to serve as the input for a 2D CNN, denoted by $h$. The CNN consists of multiple blocks of convolutions, nonlinear activations, and 2x2 nearest neighbor upsampling layers. The final output of the CNN is a complex image ($x_{t}$) corresponding to time frame $t$, with real and imaginary components represented in separate network channels.


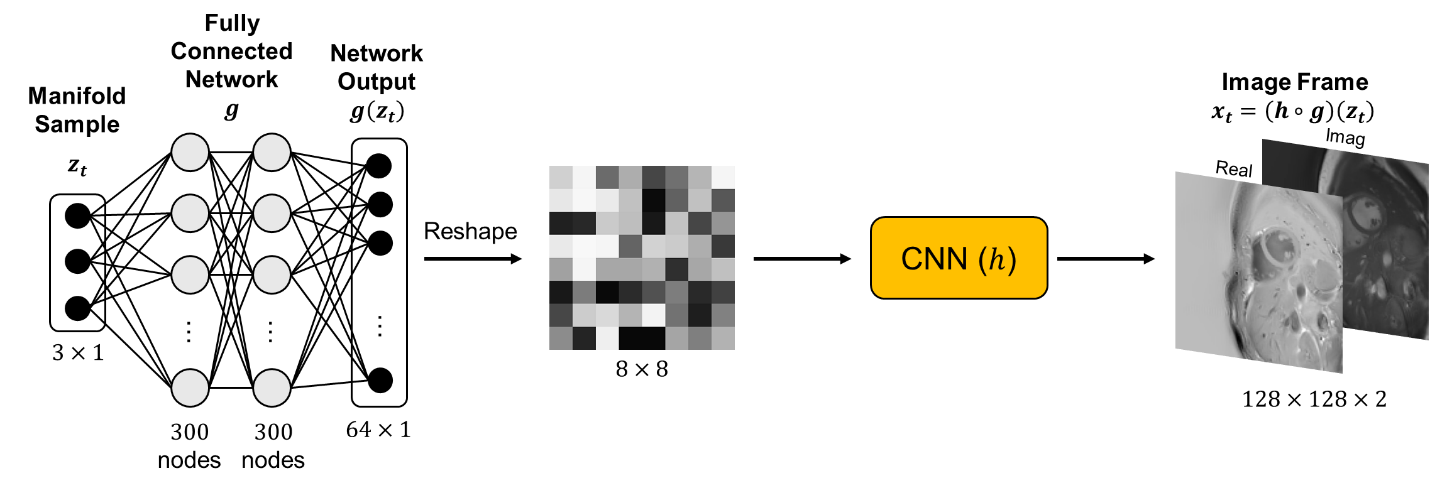


# Supporting Figure S1: *Schematic of the original Time-DIP reconstruction.*

# 2. Dimensions of the Multifrequency Manifold

Simulations were conducted to evaluate the impact of varying the dimensions of the multifrequency manifold, which is sampled for a specific time frame $t$ to produce a tensor $z_{t}$ that serves as input to the Image Reconstruction CNN. A 6-second free-breathing real-time cardiac scan was simulated using the XCAT phantom at an acceleration factor of R=8 with 6 interleaves per frame and a temporal resolution of 25 ms/frame. First, images were reconstructed using Multifrequency Time-DIP, testing different values for the height and width of $z_{t}$ of 2, 4, 8, 16, and 32. The channel dimension was kept fixed at 128. Next, reconstructions were performed using a fixed height and width of 8x8 while varying the channel dimension of $z_{t}$, testing values of 1, 2, 4, 8, 16, 32, 64, 128, 256, 512, and 1024.

**Supporting Figure S2A** shows the RMSE as a function of the height and width of $z_{t}$ (i.e., the input to the Image Reconstruction CNN). The minimum RMSE (3.4%) was achieved at a height and width of 8x8. Slightly higher errors were observed with both smaller and larger sizes, with substantially higher RMSE (7.5%) at an input size of 32x32. **Supporting Figure S2B** displays the RMSE while varying the channel dimension of the input tensor $z_{t}$. Slightly larger RMSE values (up to 4.2%) were observed when the number of channels was less than 64, and the minimum RMSE was obtained with 128 channels (3.4%). Based on these results, all subsequent experiments used dimensions of 8 (height) x 8 (width) x 128 (channels) for $z_{t}$.


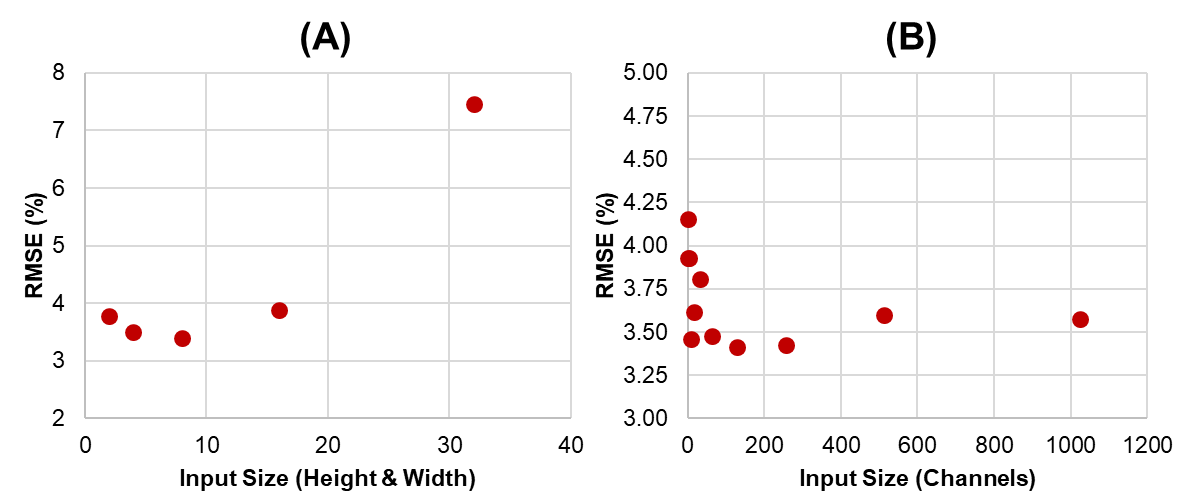


**Supporting Figure S2:** *Effect of the height, width, and channels of the input to the Image Reconstruction CNN.* RMSE values are shown for **(A)** various height and width dimensions and **(B)** different numbers of network channels for the input $z_{t}$ to the CNN.

# *3. MapNet in the Time-Dependent Deep Image Prior*

The original Time-DIP study by Yoo et al. used a fully connected network, termed MapNet, to transform the manifold to a more expressive latent space before passing this data to the CNN for image generation. This approach was found to yield lower reconstruction errors in breathheld real-time cardiac MRI when using Time-DIP with a helical manifold. Here, simulations were performed to determine whether MapNet is (a) also advantageous when using Time-DIP with a *multifrequency* manifold and (b) for *free-breathing* real-time cardiac scans. A 6-second free-breathing real-time scan was simulated using the XCAT phantom at an acceleration factor of R=8 with 238 total frames. Several Time-DIP reconstruction configurations were tested to compare reconstruction accuracy using (a) helical vs. multifrequency manifolds, both (b) with and without MapNet. Reconstruction accuracy was quantified by averaging RMSE values over all frames.

As shown in **Supporting Table S1**, the multifrequency manifold outperformed the helical manifold in all cases. Consistent with Yoo et al, the inclusion of MapNet was beneficial when paired with the helical manifold, reducing the RMSE from 5.3% to 5.1%. However, MapNet led to slightly larger errors when paired with the multifrequency manifold, increasing the RMSE from 3.5% to 3.7%. These results suggest that the multifrequency manifold alone provides sufficient representational capacity to model the temporal dynamics in free-breathing real-time cardiac scans, making the additional MapNet transformation unnecessary.

| Manifold | Network | RMSE |
| --- | --- | --- |
| Helix | CNN | 5.3% |
| Helix | MapNet & CNN | 5.1% |
| Multifrequency | CNN | 3.5% |
| Multifrequency | MapNet & CNN | 3.7% |

**Supporting Table S1:** *Simulations comparing Helix and Multifrequency Time-DIP reconstructions both with and without MapNet.*

# *4. Image Reconstruction CNN*


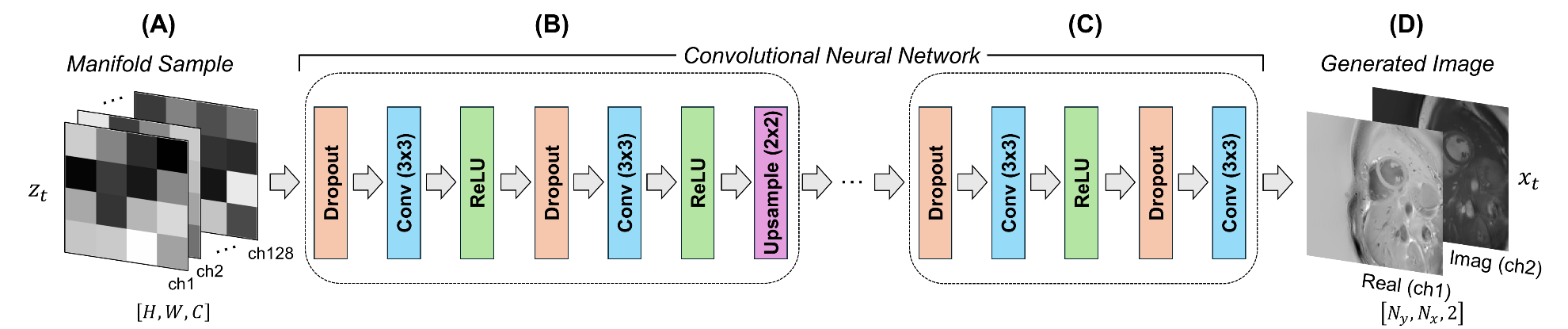


**Supporting Figure S3:** *Architecture of the Image Reconstruction CNN.* **(A)** The multifrequency manifold is sampled for a specific time frame $t$. The manifold sample $z_{t}$, which has a size of $H\times W\times C$ (height x width x channels), is processed by a CNN consisting of several blocks. **(B)** Each block includes 3x3 convolutions, ReLU activations, dropout, and 2x2 nearest neighbor interpolation (upsampling). The number of blocks in the CNN depends on the height and width of $z_{t}$ and of the reconstructed image $x_{t}$. This study required four blocks, since four upsampling steps were needed to given the dimensions of the input $z_{t}$ (height and width of 8 x 8) and the final image (128 x 128). **(C)** The last block in the CNN differs slightly from the rest, as it lacks a final activation function and does not perform upsampling. **(D)** The CNN outputs a complex-valued image, with two channels for the real and imaginary components.

# *5. Sensitivity Map CNN*


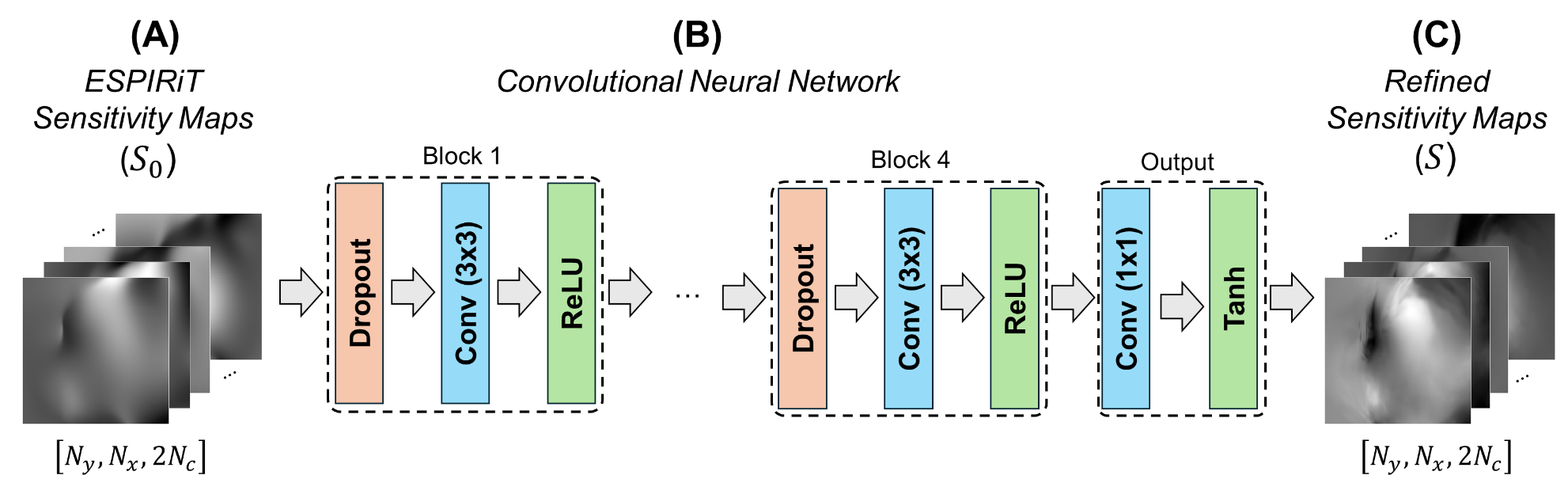


**Supporting Figure S4:** *Architecture of the CNN for coil sensitivity estimation.* **(A)** An initial estimate of the sensitivity maps is derived from time-averaged data using ESPIRiT. These maps are reshaped into a tensor of size $N_{y}\times N_{x}\times2N_{c}$, where $N_{c}$ represents the number of MRI receiver coils and the factor of 2 accounts for the real and imaginary components. **(B)** The CNN consists of four blocks, each containing a dropout layer, 3x3 convolution, and ReLU activation function. **(C)** The final output is a refined estimate of the coil sensitivity maps.

# *6. Dropout Regularization to Mitigate Overfitting*


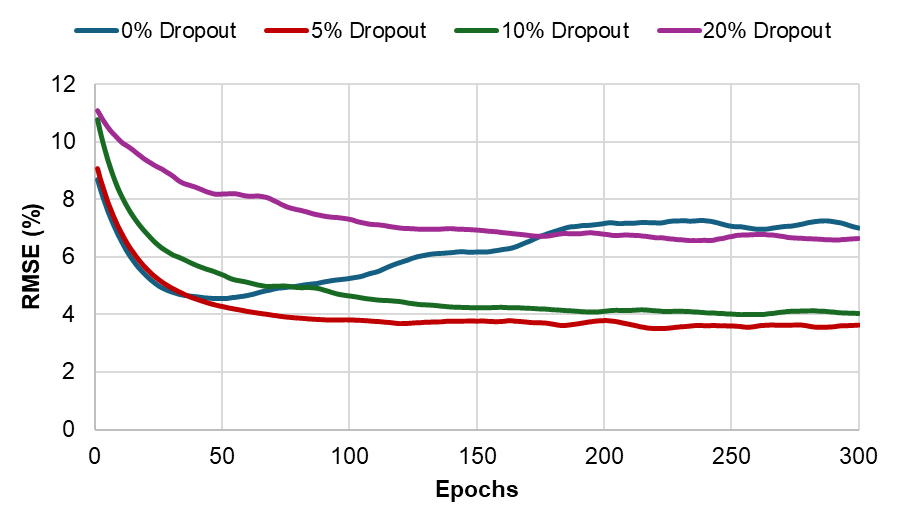
Simulations were performed to investigate the effect of training with varying levels of dropout and to guide the selection of dropout rate and number of training epochs used in subsequent experiments. The XCAT phantom was used to simulate a free-breathing ungated cardiac scan with golden angle spiral sampling at an acceleration rate of R=8 (6 interleaves/frame). Complex Gaussian noise with a standard deviation of 1% of the maximum DC signal was added to the simulated k-space data. Images were reconstructed using Multifrequency Time-DIP, trained for 300 epochs with dropout levels of 0%, 5%, 10%, and 20%. **Supporting Figure S5** plots the RMSE of the reconstructed images as a function of training epochs for each dropout level. Without dropout, the RMSE initially reaches a minimum value of 4.6% around epoch #50 but then increases as the network overfits to noise and aliasing artifacts, reaching an RMSE of 7.0% by epoch #300. When the dropout rate is too high (e.g., 20% dropout), the network converges slowly and the reconstructed images are overly smoothed, resulting in a high RMSE (6.6%) by epoch #300. The best performance is observed at a smaller dropout rate of 5%. This dropout rate stabilizes the training process, causing the RMSE to monotonically decrease and achieve a lower final RMSE of 3.5% at epoch #300.

**Supporting Figure S5:** *Simulation results showing RMSE vs. training epochs with different levels of dropout regularization.*

# *7. Variability Due to Stochastic Training*

With both Helix and Multifrequency Time-DIP, there are stochastic components in the reconstruction, including the random selection of time frames in mini-batches, random initialization of network weights, and use of a stochastic optimization algorithm (Adam). As a result, repeated reconstructions of the same dataset can yield slightly different outcomes. To quantify this variability, ten repeated reconstructions using both methods were performed using the XCAT phantom to simulate free-breathing scan with an arrhythmia (R=8, temporal resolution 25 ms/frame). This corresponds to the same dataset shown in the rightmost bar in Figure 3A of the main text. The mean and standard deviation of RMSE values across repetitions were computed, and differences between methods were assessed using a two-sample t-test. As shown in **Supporting Figure S6**, Multifrequency Time-DIP yielded significantly lower RMSE values than Helix Time-DIP (4.0$\pm$0.1% vs. 6.2$\pm$0.1%, p<0.001). The small spread in RMSE values (standard deviation of $\pm$0.1%) suggests that both methods produce consistent results despite the stochastic nature of the network training.

**Supporting Figure S6:** *Simulation results showing the distribution of RMSE values across 10 repeated reconstructions of the same dataset using Helix Time-DIP and Multifrequency Time-DIP. Asterisks indicate statistical significance (p<0.001).*

***7. Captions for Supporting Videos***

**Supporting Video S1:** Real-time spiral images (R=8, 25 ms/frame) from a healthy subject are shown at apical, mid, and basal slice positions. From left to right, each column shows the same dataset reconstructed using CS (left), Helix Time-DIP (middle), and Multifrequency Time-DIP (right) methods. This movie corresponds to the same dataset shown in Figure 5.

**Supporting Video S2:** Conventional breathheld ECG-gated cine images are presented from the same healthy subject as in Figure 5 and Supporting Video S1.

**Supporting Video S3:** Real-time spiral images (R=8, 25 ms/frame) from a patient with atrial fibrillation are shown at apical, mid, and basal slice positions. From left to right, each column shows the same dataset reconstructed using CS (left), Helix Time-DIP (middle), and Multifrequency Time-DIP (right) methods. This movie corresponds to the same dataset shown in Figure 6.

**Supporting Video S4:** Conventional breathheld ECG-gated cine images are presented from the same patient with atrial fibrillation shown in Figure 6 and Supporting Video S3. ECG mis-gating artifacts and blurring are visible particularly in the apical and basal slices. These artifacts are not seen on the real-time scans in Supporting Video S3.

**Supporting Video S5:** Real-time spiral images (R=8, 25 ms/frame) from a patient with premature ventricular contractions are shown at apical, mid, and basal slice positions. From left to right, each column shows the same dataset reconstructed using CS (left), Helix Time-DIP (middle), and Multifrequency Time-DIP (right) methods.

**Supporting Video S6:** Conventional breathheld ECG-gated cine images are presented from the same patient with premature ventricular contractions as in Supporting Video S5.

**Supporting Video S7:** Real-time spiral images from a patient with premature ventricular contractions are shown at a temporal resolution equal to a single TR (4.2 ms/frame, one spiral interleaf per frame, R=48). Images were reconstructed using CS (left), Helix Time-DIP (middle), and Multifrequency Time-DIP (right) methods.

**Supporting Video S8:** Real-time stress images at peak exercise are shown from a healthy subject pedaling inside the scanner on a supine ergometer (still images are shown in Figure 15). The real-time spiral data were binned to a temporal resolution of 17 ms/frame (R=12, 4 spiral interleaves per frame) and reconstructed using CS (left), Helix Time-DIP (middle), and Multifrequency Time-DIP (right) methods.
